# PLS regression-based chemometric modeling of odorant properties of diverse chemical constituents of black tea and coffee†

**DOI:** 10.1039/c7ra12914a

**Published:** 2018-01-09

**Authors:** Probir Kumar Ojha, Kunal Roy

**Affiliations:** Drug Theoretics and Cheminformatics Laboratory, Department of Pharmaceutical Technology, Jadavpur University Kolkata 700 032 India kunalroy_in@yahoo.com kunal.roy@jadavpuruniversity.in http://sites.google.com/site/kunalroyindia/ +91-33-2837-1078 +91 98315 94140

## Abstract

Tea and coffee are the most attractive non-alcoholic beverages used worldwide due to the odorant properties of diverse components present in these beverages. The aim of this work is to investigate the key structural features which regulate the odorant properties of constituents present in black tea and coffee using regression-based chemometric models. We have also investigated the key structural properties which create the odor difference between tea and coffee. We have employed different variable selection strategies to extract the most relevant variables prior to development of final partial least squares (PLS) models. The models were extensively validated using different validation metrics, and the results justify the reliability and usefulness of the developed predictive PLS models. The best PLS model captured the necessary structural information on relative hydrophobic surface area, heteroatoms with higher number of multiple bonds, hydrogen atoms connected to C^3^(sp^3^)/C^2^(sp^2^)/C^3^(sp^2^)/C^3^(sp) fragments, electron-richness, C–O atom pairs at the topological distance 10 and surface weighted charged partial negative surface areas for explaining the odorant properties of the constituents present in black tea. On the other hand, C–S atom pairs at the topological distance 1, C–C atom pairs at the topological distance 5, donor atoms like N and O for hydrogen bonds, hydrogen atoms connected to C^3^(sp^3^)/C^2^(sp^2^)/C^3^(sp^2^)/C^3^(sp) fragments and R–CX–X fragments (where, R represents any group linked through carbon and X represents any heteroatom (O, N, S, P, Se, and halogens)) are the key structural components captured by the PLS model developed from the constituents present in coffee. The developed models can thus be successfully utilized for *in silico* prediction of odorant properties of diverse classes of compounds and exploration of the structural information which creates the odor difference between black tea and coffee.

## Abstract 2

We investigate the key structural features regulating the odorant properties of constituents present in black tea and coffee, the most attractive non-alcoholic beverages.

## Graphical abstract

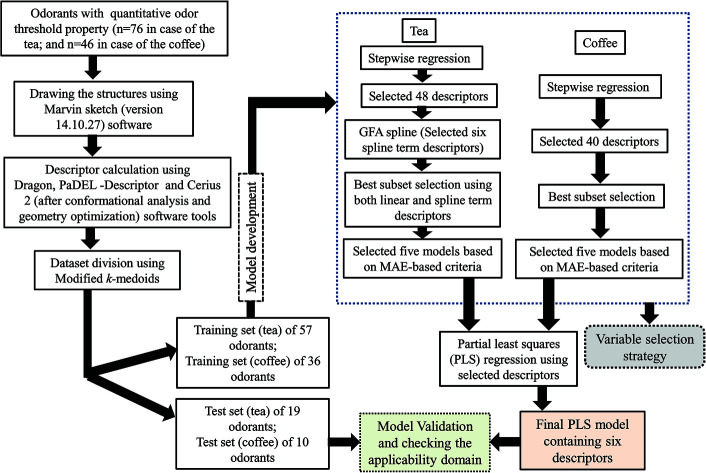

## 1. Introduction

After water, tea is the most consumed beverage worldwide amongst the non-alcoholic drinks. In 2009, the total production of tea worldwide was approximately 3.8 million metric tons.^1^ Among the total global production, China contributes 35.4% followed by India (20.6%), Kenya (8.1%), Srilanka (7.5%), Turkey (5.1%), Vietnam (4.8%), and Indonesia (4.1%).^2^ Mainly three types of tea are produced such as green tea (unfermented), oolong tea (semi-fermented) and black tea (fermented). Among these three types of tea, black tea is widely used due to its flavor. In black tea preparation, different enzymatic processes and biochemical reactions are known to occur prior to the drying process. In black tea, the key components responsible for taste are mainly polyphenols, free amino acids, caffeine, catechin, theaflavins, and thearubigins.^3–5^ Many researchers have investigated the volatile components of black tea and reported around 600 volatile compounds present in tea leaves or beverages.^6^ The purpose of fermentation in the case of black tea is to enhance the flavor of the tea. Thus, flavor is one of the most important characteristics necessary to improve the quality of tea. The flavor of tea encompasses both aroma active compounds and taste. Volatile components like aldehydes, alcohols, ketones, furans, and aromatic compounds are mainly responsible for the aroma of tea.^6^

Like tea, coffee is also an important beverage consumed worldwide in daily routine. Coffee is a relatively young beverage than tea that has been known since the 17^th^ century.^7^ It is a major source of income for many coffee producing countries like Brazil, Vietnam, Colombia, Indonesia, Ethiopia, India, Honduras, Uganda, Mexico, Guatemala, Peru, *etc.*^8^ The world wide use of this beverage is due to several factors. Among these, flavor is the main reason for its success. The final expression and perceptible results of a freshly prepared cup of coffee is due to its flavor which depends on several factors like genetic predispositions, environmental and climatic factors, harvest and post-harvest practices, sorting, grading, storage and transport, processing steps such as roasting, grinding and extraction and finally consumption practices.^7^

The odor active molecules play a crucial role to regulate the quality of both tea and coffee and make them suitable as beverages worldwide. The odor threshold (OT) presents a key attribute to all the odor active molecules. Unique characteristics of smell can help in the detection of different food and beverages for different food industries. Odor is also helpful for masking of obnoxious odor of chemicals used in different food, pharmaceuticals and cosmetic industries particularly in case of perfume and beverage industries. Thus, it might be useful to know what an odor and OT are. An odor is the impression in the brain obtained by the detection of a volatile component (mostly) at a very low concentration by odorant receptors (ORs) that is perceived by the sense of olfaction of human or other animals. The OT is the minimum concentration at which all panelists have been able to recognize the odor sensitivity which is a typical attribute of that individual compound and have been reliable in their response at all higher test concentrations. A group of fifteen observers (approx.) with working experience more than one year on analytical odor might be selected as panel members.^9^ OT can be quantified by various methodologies like GC/MS, electronic noses and measurement of electro-olfactograms for lower animals like insects,^10^ well-known psychophysical methods like triangle odor bag method,^11^ dilution-to-threshold method,^12^ scentometry,^13^ olfactometry,^14^*etc.* The value of OT may differ due to the protocols used for measurement. Thus, olfaction has emerged as an important topic of interest for researchers for many decades. It is very difficult to identify the key structural features which are essential for OT property of tea and coffee. Since there is no such modern technology which can mimic the efficiency of human nose and can characterize different types of odor with the similar sensitivity, it is useful to apply an *in silico* tool to predict OT property of odorants. Again, a small modification in the chemical structures may bring changes in odor property, *e.g.*, introduction of one or more double bonds in aliphatic alcohols or aldehydes changes the odor profile of the compounds.^15^ Thus, a proper knowledge regarding the structure–property relationship related to these odorous molecules is essential to unfold the ambiguity behind these. In this regard, quantitative structure–property relationship (QSPR)^16,17^ approach may help us to draw a correlation between structural properties and OT properties. A previous study in this direction may be cited here.^18^ The QSPR approach correlate the molecular properties with biological activities/properties/toxicities for a set of compounds by developing appropriate models, represented as numerical equations developed using different chemometric tools.

In this work, we have performed QSPR modeling of odorants present in black tea and coffee separately using their odor threshold properties to identify the key structural attributes which make these beverages attractive worldwide. We have also investigated the key structural properties which make the odor difference between tea and coffee using this *in silico* approach. The predictive QSPR models were developed in this study keeping in mind the principles of Organization for Economic Co-operation and Development (OECD) for QSPR model development.^19^

## 2. Methods and materials

### 2.1 Dataset

This work was carried out using OT property data for diverse classes (aldehyde, acid, ester, furan, sulfur containing compounds, thiols, thiophene, thiazole, furanone, ketone, norisoprenoid, phenolic compounds, pyrazine, pyridine, terpene *etc.*) of compounds present in black tea (76 compounds) and coffee (46 compounds) collected from the published literature.^7,20^ Here, we have developed two PLS models separately using the constituents present in black tea and coffee. The details of the datasets are presented in Tables S1 and S2.† The odor threshold (OT) of compounds is expressed in mmol kg^−1^ in case of black tea and in μmol kg^−1^ in case of coffee. For development of QSPR models, the OT values are taken in the negative logarithmic scale [log(1/OT)] leading to *Y* ranges from −0.93487 to 7.677402 (in case of black tea) and −1.73629 to 5.532415 (in case of the coffee). Note that in case of the tea dataset, the initial modelling analysis identified one compound as potential outlier (high residual value). Thus, the final PLS model was developed using 76 components present in tea.

### 2.2 Descriptor calculation

All the structures were drawn using Marvin sketch software (http://www.chemaxon.com). The descriptors were calculated using three software tools namely Dragon software version 6,^21^ PaDEL-descriptor (http://www.yapcwsoft.com/dd/padeldescriptor) software and Cerius 2 version 4.10 software.^22^ Constitutional indices, ring descriptors, connectivity indices, functional group count, atom centered fragments, atom type E-state indices and 2D atom pairs were calculated using Dragon software while extended topochemical atom (ETA) indices were calculated using PaDEL-descriptor software. All the molecules were exported to Cerius 2 software version 4.10 (ref. 22) for conformer generation using the ‘optimal search method’. Geometry optimized molecules were used to calculated all 3D descriptors. Thus, in this work, we have used a pool of both 2D and 3D descriptors for development of the final models. Descriptors are “numerical values associated with chemical constitution for correlation of chemical structure with various physical properties, chemical reactivity or biological activity”. From the total pool of descriptors, those having constant and near constant values (standard deviation less than 0.0001) of the variables, descriptors with at least one missing value, descriptors with all missing values and descriptors with (absolute) pair correlation larger than or equal to 0.95 were excluded from the initial pool of descriptors.

### 2.3 Division of the dataset: selection of training and test sets

Considering the importance of dataset division during predictive model development,^23^ we have employed a clustering technique, “Modified *k*-medoids”,^24^ using a tool developed in our laboratory (http://teqip.jdvu.ac.in/QSAR_Tools/DTCLab). Seven clusters were generated in case of tea components and four clusters were generated in case of coffee components based on the properties available for the respective dataset components. For the selection of training and test sets, we have taken approximately 25% compounds from each cluster randomly for the test set (19 compounds in case of the tea dataset and 10 compounds for the coffee dataset) and remaining 75% compounds for the training set (57 compounds and 36 compounds in case of the tea and coffee datasets respectively). The training set was used to develop the QSPR model that was subsequently validated by the test set compounds.

### 2.4 Descriptor selection and model development

We have performed stepwise regression using the whole pool of descriptors for selection of the descriptors. After the first run of stepwise regression, we have removed the selected descriptors and rerun stepwise regression using remaining pool of descriptors. In this way, we have selected 48 descriptors in case of the tea and 40 descriptors in case of the coffee dataset. In case of the tea dataset, we have developed a few Genetic Function Approximation (GFA)^25^ models using both linear and spline options employing Cerius 2 software and selected some spline term descriptors and clubbed with the previously selected descriptors from stepwise regression (total 54 descriptors). After that, we have performed the best subset selection for development of models using a software developed in our laboratory (http://teqip.jdvu.ac.in/QSAR_Tools/DTCLab). In case of the coffee dataset, we have performed the best subset selection using only the selected descriptors obtained from stepwise regression. Note that in this case also we tried to apply GFA for obtaining spline terms; however, no significant terms were obtained in the derived models and thus the GFA models were discarded in case of modelling of the coffee data set. In both cases, we have developed six descriptor models. From these developed models, we have chosen the best five models based on mean absolute error (MAE) based criteria for the test set.^26^ Finally, we have run PLS using the descriptors obtained from these five models. Finally, we have developed six descriptor PLS models in both cases (tea and coffee data sets).

The best subset selection was performed using a software tool developed in our laboratory (http://teqip.jdvu.ac.in/QSAR_Tools/DTCLab) in order to optimize the best descriptor combinations from the reduced pool of descriptors (in case of tea, both spline and linear descriptors used; in case of coffee, only linear descriptors used). We have selected the best five multiple linear regression (MLR) models obtained from six descriptor combinations based on the MAE-based criteria^26^ of the validation sets.

### 2.5 Statistical analyses and chemometric tools employed

The chemometric tools namely stepwise regression,^27^ genetic methods (GFA)^25^ and best subset selection were used for selection of variables, while the final models were developed using the PLS methodology.^28^

#### 2.5.1 Stepwise regression

In this technique,^27^ a multiple-term linear equation is built step by step where an initial model is recognized first, and then this is repeatedly altered by adding or removing a predictor variable based on the “stepping criteria”. The stepwise regression method is a combination of the forward selection and backward elimination approaches where testing at each stage for variables to be included or excluded. In case of forward selection, one initially starts with no variables in the model and then trying to find out the ‘statistically significant’ variables one by one and including them in the model. On the other hand, in case of backward elimination, one starts with all the candidate variables and testing and deleting them one by one which are statistically insignificant. In this work, we have employed the “stepping criteria” *F* = 4 to enter and *F* = 3.9 to remove. The criteria “*F* to Enter” and “*F* to Remove” verify how significant or insignificant the role of a variable is in the regression equation, respectively for adding the variable to the equation and removing the variable from the equation. The *F* value indicates the square of the *t* value of the incoming variable which signifies the corresponding regression coefficient.

#### 2.5.2 Genetic function approximation (GFA)

The GFA algorithm (Rogers and Hopfinger)^25^ is a statistical tool which evolved from the knowledge of Holland's genetic algorithm (1975)^29^ and Friedman's multivariate adaptive regression splines (MARS) algorithm.^30^ In GFA, multiple models are generated instead of a single model (unlike stepwise regression), and the best model can be selected based on the fitness and predictive potential of the model. In this work, the GFA models were developed using the software Cerius 2 4.10 version^22^ applying both linear and spline options. The spline terms, designated by angular bracket (〈〉, chevrons), consider some aspect of nonlinearity. In GFA, descriptors are selected randomly to develop an initial population of equations followed by cross over between those pairs of equations. The model quality is judged by a fitness function or “Lack of Fit (LOF)” score. The quality of the models and the LOF score are inversely proportional, *i.e.*, model quality will increase with a decrease in the LOF value. Genetic cross-over operation is repeatedly performed after the initial rating of the models based on the LOF score. In the cross-over operation, first, two good quality models are preferred as parents and each parent is randomly cut into two pieces, and cross-over is done between two pieces taking one from each parent, and finally a new model (daughter model) is generated. In this way, good combinations of genes are discovered after many mating step (genetic cross-over) and spread through the population. To develop the GFA models, we have assigned some settings like mutation probabilities (kept at 50% with 5000 iterations), smoothness parameters (kept at 1.00), initial equation length value, *i.e.*, number of descriptors (was set to four) and finally, no fixed length for the final equations. Note that, in this work, we have used GFA algorithm only for selection of important descriptors but not the development of the final model.

#### 2.5.3 Partial least squares (PLS)

PLS is a generalization of regression which is more appropriate when the matrix of predictors has more variables than observations. This technique is also suitable in such cases when there is some intercorrelation among the *X*-variables. It is used to find out the fundamental relations between *X* and *Y* matrices, *i.e.*, a latent variable approach to modeling the covariance structures in these *X* and *Y* spaces. PLS allows to construct larger QSAR equations by avoiding overfitting and eliminating most variables. PLS is statistically more robust than MLR because standard regression will fail in such cases.^28^ To obtain the optimum number of latent variables, PLS is normally used in combination with cross-validation which ensures that the developed models are selected based on their ability to predict the data rather than to fit the data.^31^ In this work, we have developed the PLS model employing the leave-one-out (LOO) cross-validation technique for selection of optimum number of latent variables.

**Fig. 1:**
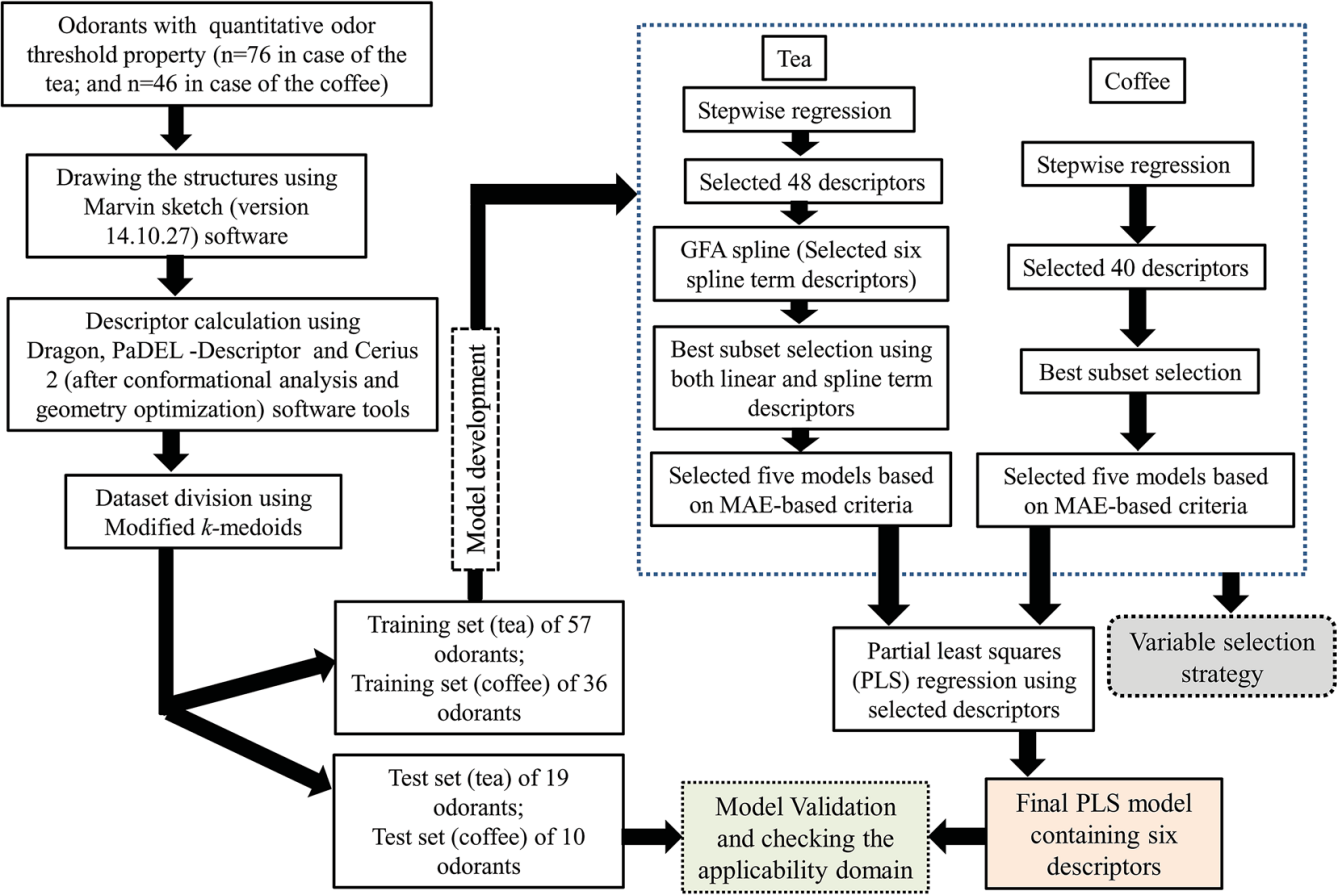
Schematic representation of the steps involved in the development of final PLS models.

The steps involved to develop the final PLS model is illustrated schematically in Fig. 1.

### 2.6 Statistical validation parameters

In this work, we have determined various statistical metrics corresponding to various validation strategies in order to justify the reliability and usefulness of the developed predictive models. The computed validation metrics for the PLS^28,32–40^ models have been explicitly tabulated and defined in Table S3 in ESI.† In addition to the classical validation parameters like leave-one-out cross-validated correlation coefficient (*Q*^2^), *R*_pred_^2^, *Q*_F2_^2^, concordance correlation coefficient (CCC) *etc.*, we have also checked *r*_m_^2^ metrics and mean absolute error (MAE) based criteria for the external set for better understanding of the quality of predictions.^26^ The final PLS models were also validated using an additional randomization test^41^ through randomly reordering (100 permutations) the *Y*-variable (log(1/OT)) (by keeping *X*-matrix intact) using SIMCA-P software^42^ to ensure that the model was not developed by any chance. Each and every randomization and consequent PLS run analysis generates a new set of *R*^2^ and *Q*^2^ values. These values are plotted against the correlation coefficient between the original *Y*-values and the permuted *Y*-values. The developed model is considered to be valid if the parameters *R*_int_^2^ and *Q*_int_^2^ are less than 0.4 and 0.05 respectively. We have also checked the acceptability of the final PLS models using external validation criteria proposed by Golbraikh and Tropsha.^43^

### 2.7 Applicability domain (AD)

We have also checked the applicability domain of the developed models to ensure that the test molecules are within the region of chemical space defined by the training set employing a DModX (distance to model X) approach^28^ at 99% confidence level using SIMCA-P software.^42^ The AD of QSAR model represented by the response and the chemical structure space which is characterized by the molecular properties of the training set molecules only. The developed QSPR models are able to predict the newly designed compound properly when the molecule lies within the region of chemical space of the training set molecules.

### 2.8 Software used

Marvin sketch (version 14.10.27) software (http://www.chemaxon.com/) was used to draw the chemical structures. Three software tools namely Dragon version 6,^21^ Cerius 2 (version 4.0)^22^ and PaDEL-descriptor (http://www.yapcwsoft.com/dd/padeldescriptor) software were used to calculate the molecular descriptors. Cluster analysis was performed by employing modified *k*-medoid (http://teqip.jdvu.ac.in/QSAR_Tools/DTCLab) software developed in our laboratory. In order to optimize the best descriptor combinations from the reduced descriptor pool, we have run best subset selection using a software developed in our laboratory (http://teqip.jdvu.ac.in/QSAR_Tools/DTCLab). The stepwise regression and PLS analysis were performed by using MINITAB software (version 14.13).^44^ SIMCA-P software^42^ was used to perform PLS model randomization, variable importance plot, score plot, regression coefficient plot and loading plot.

## 3. Results and discussion

We have developed separately, PLS models of odor active compounds present in tea (eqn (1)) and coffee (eqn (2)) using odor threshold (OT) property in the form log(1/OT). We have validated the PLS models using various statistical parameters which are summarized in Table 1. The statistical results suggested that both the models are acceptable. The MAE based criteria in case of external sets of both the models were found to be “moderate” indicating acceptability of the models. We have also validated the models using Golbraikh and Tropsha's criteria and the results are depicted in Table 2. Based on this criterion also, models are acceptable. We have also performed Y-randomization test using SIMCA-P software where the response variable (log(1/OT)) was reordered randomly (100 permutations) and the intercepts of both *R*^2^ and *Q*^2^ values were checked. The Y-randomization test was performed to verify whether the models are obtained by any chance or not. The randomization results (*R*_int_^2^ < 0.4 and *Q*_int_^2^ < 0.05) suggested that the models are not obtained by any chance and the results are depicted in Fig S1 and S2.†

**Table 1:** Statistical quality and validation parameters of the final PLS models (black tea and coffee)

Dataset	Model type	Descriptors	*R* ^2^	*R*_a_^2^	*Q* ^2^	LV	*s*	*R*_pred_^2^	*Q*_F2_^2^	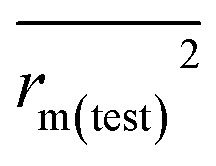	Δ*r*_m(test)_^2^	CCC	MAE based criteria (test)
Black tea	PLS model	H-049, ETA_Eta_F, ETA_BetaP_ns, Jurs-WNSA-3, F10[C–O], 〈Jurs-RASA-0.767154〉	0.616	0.578	0.534	5	1.112	0.608	0.586	0.536	0.152	0.791	Moderate
Coffee	PLS model	C-029, H-049, F05[C–C], nHDon, B01[C–S], ETA_Eta	0.722	0.696	0.639	3	1.068	0.781	0.781	0.777	0.101	0.905	Moderate

**Table 2:** Results of the final PLS models (black tea and coffee) obtained according to Golbraikh and Tropsha's criteria

		Parameters	PLS model	Remarks	Threshold value
Black tea	1	*r* ^2^	0.648	Passed	*r* ^2^ > 0.6
	2	[(*r*^2^ − *r*_0_^2^)/*r*^2^]	0.015626143	Passed	<0.1
		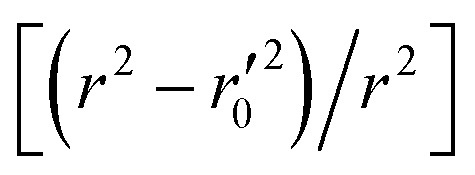	0.116611832	Passed	
	3	*k*	0.9252	Passed	0.85 < *k* or *k*′ < 1.15
		*k*′	1.0337		
Coffee	1	*r* ^2^	0.837	Passed	r^2^ > 0.6
	2	[(*r*^2^ − *r*_0_^2^)/*r*^2^]	0.015552055	Passed	<0.1
		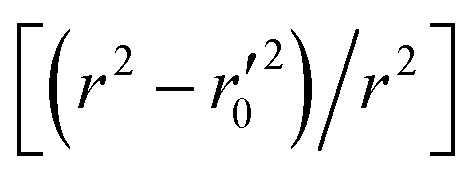	0.000415929	Passed	
	3	*k*	0.8815	Passed	0.85 < *k* or *k*′ < 1.15
		*k*′	1.0561		

### 3.1 PLS model developed from odorants present in black tea



1

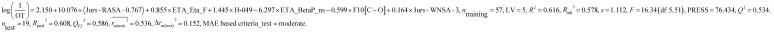




The above PLS model (eqn (1)) is derived from five latent variables and six descriptors which are the key structural features linked to^45^ tea aroma. Using the variable importance plot (VIP)^46^ (Fig. S3†), the significance level of the descriptors was found to be in the following order: 〈Jurs-RASA-0.767〉, ETA_Eta_F, H-049, ETA_BetaP_ns, F10[C–O] and Jurs-WNSA-3. The values of the descriptors appearing in eqn (1) for different compounds are shown in Table S4.†

The highest significant descriptor, 〈Jurs-RASA-0.767〉, involves the relative hydrophobic surface area which is calculated by total hydrophobic surface area divided by total molecular solvent accessible surface area. The positive regression coefficient (Fig. S4†) of this spline term descriptor indicates that the numerical value of Jurs-RASA should be more than the knot value of 0.767 for a higher odorant property. It has been found that the compound no. 47 (linalool), 62 (2-undecanone) and 68 (β-damascenone) show higher range of odorant property as their corresponding Jurs-RASA value is more than 0.767 while compound no. 11 (propanoic acid), 12 (2-methyl propanoic acid) and 17 (furfural) show lower range of odorant property as their numerical value of Jurs-RASA is less than the knot value of 0.767. Note that 10 out of 57 odorants present in the training set [compound no. 8 (acetic acid), 11 (propanoic acid), 12 (2-methyl propanoic acid), 15 (butanoic acid), 17 (furfural), 18 (3-methyl butanoic acid), 19 (2-methyl butanoic acid), 54 (octanoic acid), 61 (nonanoic acid), and 69 (vanillin)] have numerical values of Jurs-RASA lower than the knot value of 0.767. These compounds are mostly acids in nature except furfural (aldehyde) and most of them share unpleasant odors like sweaty, sour, vinegary, pungent, rancid, *etc.* Thus, from this descriptor, it can be concluded that hydrophobic surface area plays a crucial role to regulate the odorant property of black tea components.

The second highest significant descriptor ETA_Eta_F, the functionality index, gives a measure of the number of heteroatoms and multiple bonds. This descriptor contributes positively towards the odorant property as indicated by positive regression coefficient (Fig. S4†). Thus, the compounds bearing any heteroatoms or more number of multiple bonds as found in compound no. 44 ((*E*,*E*)-3,5-octadien-2-one) (one oxygen atom and three double bonds), 68 (β-damascenone) (one oxygen atom and four double bonds) and 71 (α-ionone) (one oxygen atom and three double bonds) have higher odorant property. Again, the compounds having lower number of heteroatoms or lower number of multiple bonds show lower range of odorant property as in case of compound no. 2 (acetone) (one oxygen atom and one double bond), 5 (1-butanol) (one oxygen atom but no double bond), 8 (acetic acid) (two oxygen atom and one double bond) and 22 ((*E*)-2-hexen-1-ol) (one oxygen atom and one double bond). Thus, compounds containing any polar group are influential to enhance the odorant property of tea. From this descriptor, it can be interpreted that the molecules having heteroatoms with higher number of multiple bonds are influential for odorant property of black tea.

The third highest significant atom-centred fragment descriptor, H-049, indicates H atom attached to C^3^sp^3^, C^2–3^sp^2^, C^1–3^sp. The subscript represents hybridization and the superscript is its formal oxidation number. The formal oxidation number of a carbon atom equals the sum of the formal bond orders with electronegative atoms. The positive regression coefficient (Fig. S4†) of this descriptor indicates that compounds bearing this fragment have higher odorant property as shown in compound no. 26 ((*Z*)-4-heptenal) (one –CHO group), 51 ((*E*,*Z*)-2,6-nonadienal) (one –CHO group), 56 ((*E*,*E*)-2,4-nonadienal) (one –CHO group) and 70 (dodecanal) (one –CHO group) (these compounds contain one hydrogen atom attached with a sp^2^ carbon atom which is attached with one oxygen atom) where as compound no. 2 (acetone), 8 (acetic acid), 11 (propanoic acid) and 12 (2-methyl propanoic acid) show lower odorant property as these compounds are devoid of this fragment. From this observation, it can be concluded that the molecules containing hydrogen atom connected with C^3^(sp^3^)/C^2^(sp^2^)/C^3^(sp^2^)/C^3^(sp) fragments connected with a heteroatom are influential for odorant property of black tea.

The fourth highest significant descriptor, ETA_BetaP_ns, gives a measure of electron-richness of the molecules relative to the molecular size. Therefore, electron-richness (unsaturation) relative to the molecular size of molecule is an important parameter to regulate the odorant property of tea. The negative regression coefficient (Fig. S4†) of this parameter indicates that electron density of molecules should be lower for increasing the odorant property of black tea as found in the compound no. 31 (1-heptanol), 62 (2-undecanone) and 70 (dodecanal) whereas the compounds with high electron density show lower range of odorant property as shown in compound no. 17 (furfural), 41 (benzyl alcohol) and 50 (2-phenyl ethanol). Thus, from this descriptor, it can be concluded that the molecules should be less electron-rich for higher odorant property.

The next significant descriptor, F10[C–O], a 2D atom pair descriptor, indicates the frequency of C–O bond at the topological distance 10. The negative regression coefficient (Fig. S4†) of this descriptor indicates that presence of higher number of C–O bonds at the topological distance 10 is detrimental for odorant property of black tea as shown in compound no. 67 (decanoic acid), 74 (dodecanoic acid) and 76 (tetradecanoic acid) (all these compounds contain two C–O bonds at topological distance 10) and *vice versa* as shown in compound no. 26 ((*Z*)-4-heptenal), 51 ((*E*,*Z*)-2,6-nonadienal) and 68 (β-damascenone) (these compounds have no C–O bond at topological distance 10).

The least significant descriptor, Jurs-WNSA-3, is the surface weighted charged partial negative surface areas. It is the partial negative surface area (PNSA-3) multiplied by the total molecular solvent-accessible surface area (SASA) and divided by 1000, *i.e.*
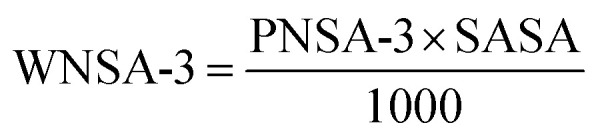


Partial negative surface area (PNSA-3) is the sum of the product of solvent-accessible surface area multiplied by partial charge for all negatively charged atoms.

From the eqn (1), it can be observed that partial charge for all negatively charged atoms (PNSA-3) may enhance the odorant property of tea components. The positive regression coefficient (Fig. S4†) of Jurs-WNSA-3 indicates that the odorant property of black tea components is directly correlated with surface weighted charged partial negative surface areas. Thus, the odorant property will increase with an increase in the numerical value of surface weighted charged partial negative surface areas (as shown in compound no. 51 ((*E*,*Z*)-2,6-nonadienal), 62 (2-undecanone) and 70 (dodecanal)) and decrease with a decrease the numerical value of surface weighted charged partial negative surface areas (as shown in compound no. 2 (acetone), 4 (ethyl acetate) and 41 (benzyl alcohol)).

**Fig. 2:**
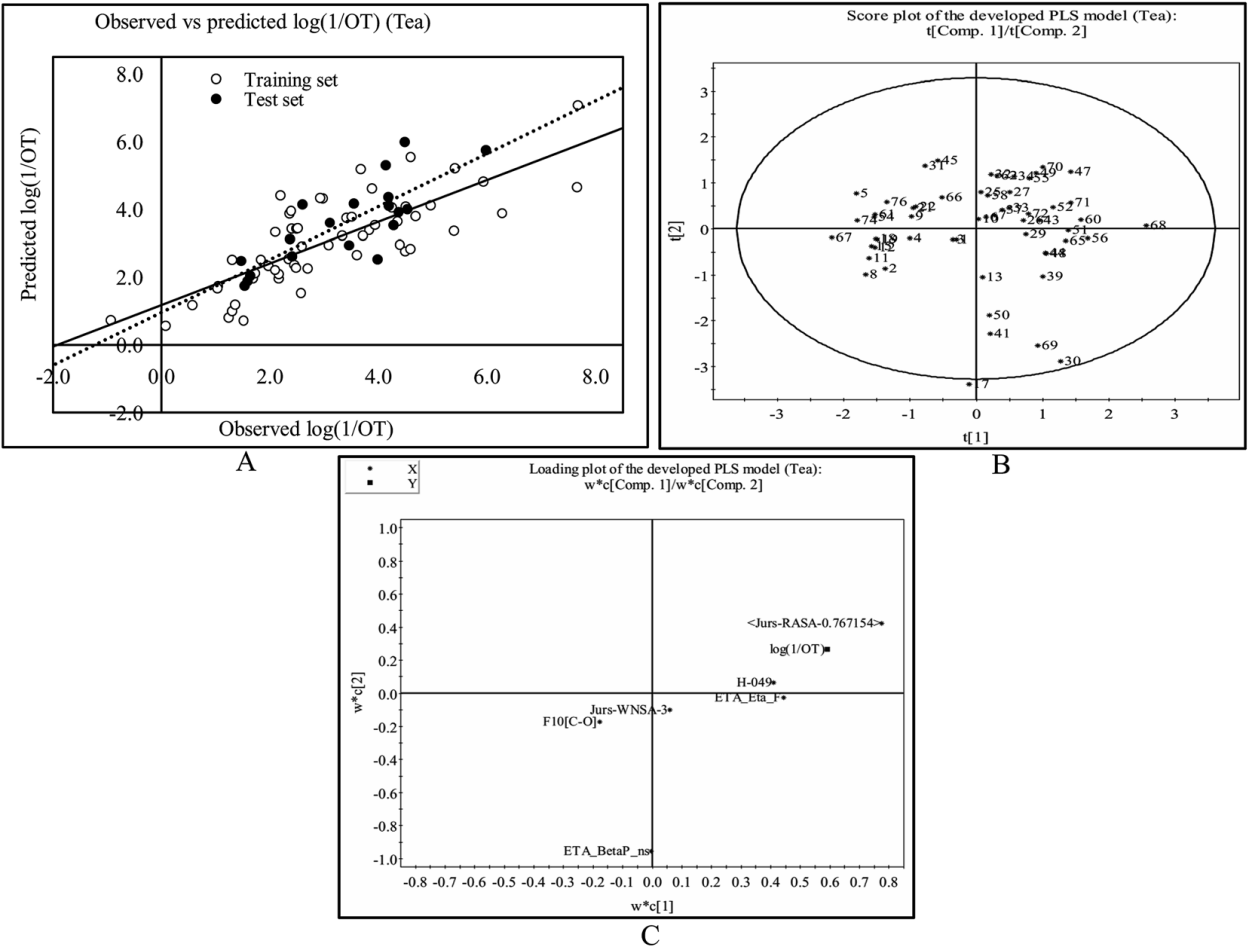
The PLS model developed from the constituents present in black tea: (A) the scatter plot of the observed and the predicted values of odorant property [log(1/OT)] for the final PLS model. The dashed line indicates the best fit line based on test set compounds and the solid line indicates the best fit line based on the training set compounds. (B) The PLS score plot of the training set compounds using the developed PLS model. (C) The loading plot of the model descriptors and dependent variable (log(1/OT)).

The observed and predicted odorant properties of molecules present in black tea are presented graphically in Fig. 2A.

#### 3.1.1 Score plot of the PLS model^47^

Score plot is important to explore the distribution of molecules in the latent variable space. The scores obtained from first two components t1 and t2 are only plotted here to see the distribution of molecules and also check any outliers are present in the dataset or not. If any compound is positioned outside the ellipse (at 99% significance level), then we can consider that compound as an outlier. In the score plot, the ellipse represents the applicability domain of the PLS model developed by using black tea components as defined by Hotelling's T^2^. Hotelling's T^2^ is a multivariate generalization of Student's *t*-test.^48^ We can also identify the outliers from this plot. Fig. 2B shows that compound no. 27 (heptanal), 34 (2-octanone), 47 (linalool), 49 (nonanal), 52 ((*E*)-2-nonenal) and 70 (dodecanal) are situated in the upper right hand corner bearing similar properties whereas the compounds which are far apart from each other like those situated in the lower left hand corner (compound no. 2 (acetone), 4 (ethyl acetate), 8 (acetic acid) and 11 (propanoic acid)) and upper right hand corner (34 (2-octanone), 47 (linalool), 49 (nonanal) and 70 (dodecanal)) represent dissimilar compounds. It has also been found from the Fig. 2B that compound number 17 (furfural) is situated outside the ellipse and indicated as an outlier.

#### 3.1.2 Loading plot of the PLS model^47^

Loading plot gives us some idea about the relationships between the *X*-variables and *Y*-variables. The loading plot deals with the impact of model descriptors on the odorant property of the molecules present in tea and also to identify the similar and dissimilar descriptors among them. Here, we have used first two components for developing the loading plot. The variables contributing similar type of information are positioned like a cluster or group. The variables which are situated far apart from the plot origin are considered to have a strong impact on the developed model. The sign of the PLS loading also provides essential information regarding the correlation among the variables. From the loading plot (Fig. 2C), we have found that the spline term descriptor 〈Jurs-RASA-0.767〉 and H-049 descriptor are directly correlated with the odorant property due their closeness to the *Y*-variables (log(1/OT)) while the descriptors Jurs-WNSA-3, F10[C–O] and ETA_BetaP_ns are inversely correlated with the odorant property of the molecules as these descriptors are situated opposite side of the *Y*-variable. From this plot, it can be interpreted that 〈Jurs-RASA-0.767〉, ETA_Eta_F and H-049 descriptors are influential to the odorant property of the compounds present in black tea as shown in compound no. 26 ((*Z*)-4-heptenal), 44 ((*E*,*E*)-3,5-octadien-2-one), 47 (linalool), 51 ((*E*,*Z*)-2,6-nonadienal), 56 ((*E*,*E*)-2,4-nonadienal), 62 (2-undecanone), 68 (β-damascenone) and 70 (dodecanal) while H-049 and nHDon descriptors are detrimental towards the odorant property as shown in compound no. 2 (acetone), 4 (ethyl acetate), 17 (furfural), 41 (benzyl alcohol), 50 (2-phenyl ethanol), 67 (decanoic acid), 74 (dodecanoic acid) and 76 (tetradecanoic acid). The loading plot also showed that all the *X*-variables are loaded strongly in the model and divided into two groups. The first group is formed by 〈Jurs-RASA-0.767〉, H-049, Jurs-WNSA-3, F10[C–O] and ETA_BetaP_ns descriptors while the second group is formed by only ETA-Eta_F descriptor which have a positive impact towards the odorant property of the constituents present in black tea but this descriptor is not similar to the other five descriptors.

### 3.2 PLS model developed from odorants present in coffee

This PLS model (eqn (2)) is derived from three latent variables obtained from six descriptors. Based on the variable importance plot (VIP) (Fig. S5†),^46^ the significance level of the descriptors was found to be in the following order: B01[C–S], ETA_Eta, F05[C–C], nHDon, H-049 and C-029.2
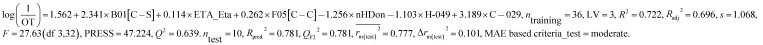


The values of the descriptors appearing in eqn (2) for different compounds are shown in Table S5.†

The most significant descriptor, B01[C–S], a 2D atom pair descriptor, indicates the presence/absence of C–S bond at the topological distance 1. The positive regression coefficient (Fig. S6†) of this descriptor indicates that the frequency of C–S fragment at the topological distance 1 is directly proportional to the odorant property of compounds present in coffee. A higher number of this fragment correlates to higher odorant property of compounds as observed in compound no. 13 (dimethyl trisulfide), 16 (3-mercapto-3-methylbutyl formate), 18 (2-methyl-3-furanthiol) and 19 (3-methyl-2-butene-1-thiol) (each compound containing one such fragment), while a lower numerical value of this descriptor correlates to lower odorant property of odorants present in coffee as observed in compound no. 35 (2,3-dimethylpyrazine), 42 (2-methoxy-3-isopropylpyrazine) and 43 (pyridine) (containing no such fragment). Thus, presence of this fragment at the topological distance 1 is influential to enhance the odorant property of compounds present in coffee.

The second highest significant descriptor, ETA_Eta, represents the topological environment of molecules. This descriptor contributed positively towards the odorant property as indicated by the positive regression coefficient (Fig. S6†). Thus, the higher numerical value of this descriptor is influential to enhance the odorant property of odorants as shown in compound no. 16, 30 and 37 and *vice versa* in case of compound no. 4 (acetaldehyde), 12 (5-methyl-2-furancarboxyaldehyde) and 43 (pyridine).

The third highest significant descriptor, F05[C–C], a 2D atom pair descriptor, indicates the frequency of C–C bond at the topological distance five. This descriptor has a positive contribution towards the odorant property of coffee components as indicated by positive regression coefficient (Fig. S6†). Thus, the compounds bearing this bond at the topological distance five show higher range of odor threshold property as evidenced by the compounds 30 ((*E*)-β-damascenone) (frequency of such atom pair at topological distance five is eight), 37 (2,3-diethyl-5-methylpyrazine) (frequency of such atom pair at topological distance five is five) and 41 (2-methoxy-3-isopropylpyrazine) (frequency of such atom pair at topological distance five is five) while the compounds (compound no. 29 (2,3-pentanedione), 35 (2,3-dimethylpyrazine) and 43 (pyridine)) containing no such bonds at topological distance five show poor odorant property.

The fourth highest significant descriptor, nHDon, a functional group count descriptor, indicates the number of donor atoms for H-bonds (N and O). This descriptor has a negative contribution (negative regression coefficient) (Fig. S6†) towards the odorant property of coffee components. This indicates that propensity of hydrogen bonding of coffee components is detrimental for enhanced odorant property of molecules. As for example, compound no. 22 (2-ethyl-4-hydroxy-5-methyl-3(2*H*)-furanone), 32 (4-ethyl guaiacol) and 34 (vanillin) show lower range of odorant property as these compounds containing one hydrogen bond donor atom each whereas compound no. 18 (2-methyl-3-furanthiol), 19 (3-methyl-2-butene-1-thiol) and 21 (dihydro-2-methyl-3(2*H*)-furanone) show higher range of odorant property as these compounds contain no such (N, O) donor atoms for hydrogen bonding.

The next highest significant atom-centred fragments descriptor, H-049, indicates H atom attached to C^3^sp^3^, C^2–3^sp^2^, C^1–3^sp as discussed previously in eqn (1). The negative regression coefficient (Fig. S6†) of this descriptor indicates that this fragment has a negative impact towards the odorant property of coffee components. Interestingly, this fragment has a positive contribution towards the odorant property in case of the equation obtained from black tea components. Thus, this fragment contributed oppositely towards the odorant property in case of tea and coffee. It has been found that compound no. 19 (3-methyl-2-butene-1-thiol), 21 (dihydro-2-methyl-3(2*H*)-furanone), 27 (1-octen-3-one) and 30 ((*E*)-β-damascenone) (no such fragment) show higher range of odorant property whereas compound no. 35 (2,3-dimethylpyrazine) (two H–CH–N fragments), 42 (2-methoxy-3-isopropylpyrazine) (three H–CH–N fragments) and 43 (pyridine) (two H–CH–N fragments) show lower range of odorant property due to the presence of this fragment.

The least significant descriptor, C-029, atom-centred fragments, indicates the fragment R–CX–X, where R represents any group linked through carbon and X represents any heteroatom (O, N, S, P, Se, and halogens). The positive regression coefficient (Fig. S6†) of this descriptor indicates that presence of this fragment in coffee component may enhance the odorant property as shown in compound no. 40 (2-methoxy-3,5-dimethylpyrazine) and 41 (2-methoxy-3-isopropylpyrazine) while the compounds without these fragments (as shown in compound no. 8 (3-methylbutyric acid), 22 (2-ethyl-4-hydroxy-5-methyl-3(2*H*)-furanone) and 29 (2,3-pentanedione)) show poor odorant property. Therefore, the components present in coffee bearing these R–CX–X fragments play a crucial role to regulate the aroma properties which make it suitable ideal beverages worldwide.

**Fig. 3:**
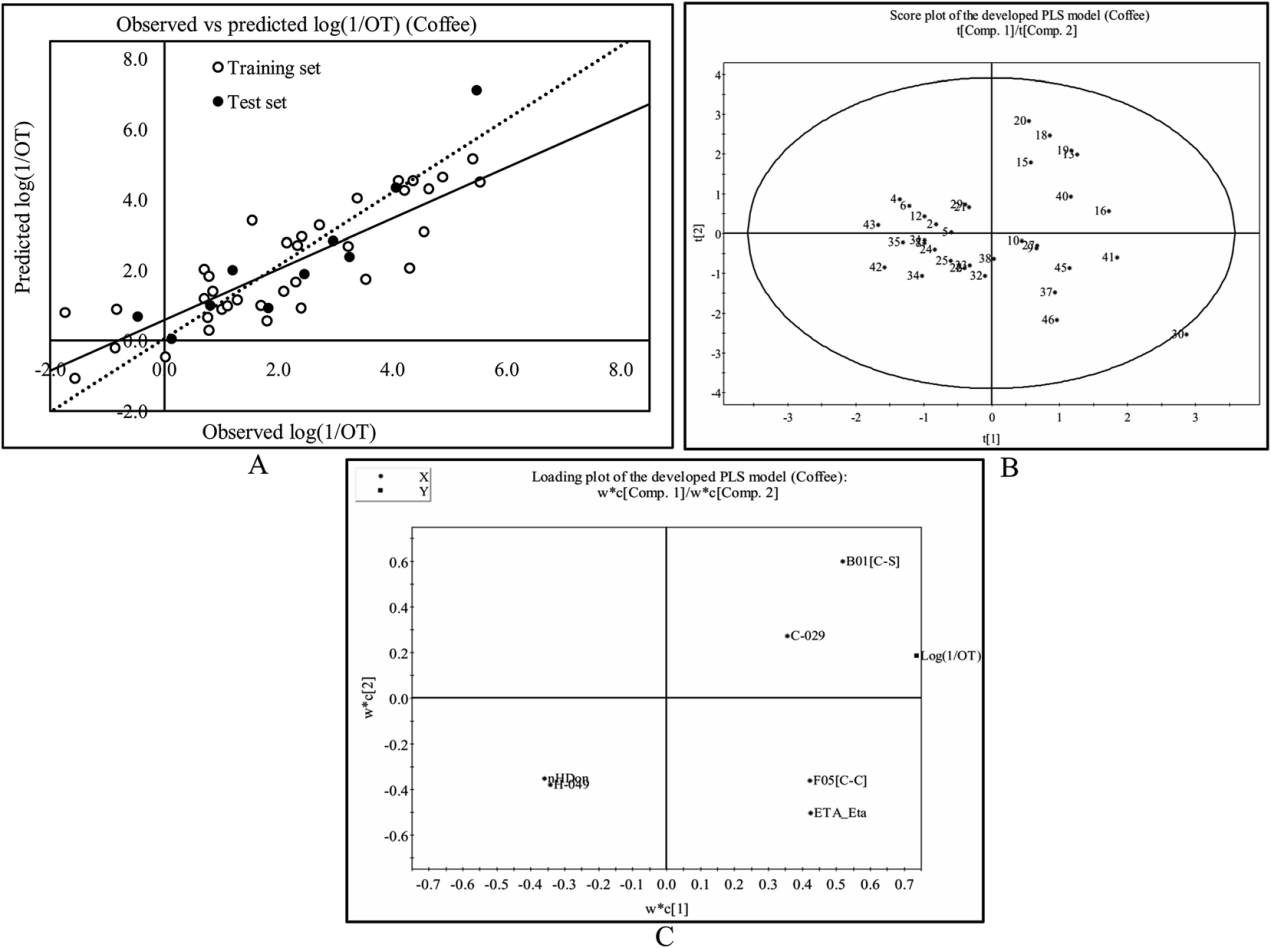
The PLS model developed from the constituents present in coffee: (A) the scatter plot of the observed and the predicted values of odorant property [log(1/OT)] for the final PLS model. The dashed line indicates the best fit line based on test set compounds and the solid line indicates the best fit line based on the training set compounds. (B) The PLS score plot of the training set compounds using the developed PLS model. (C) The loading plot of the model descriptors and dependent variable (log(1/OT)).

The observed and predicted odorant properties of molecules present in coffee are presented graphically in Fig. 3A.

#### 3.2.1 Score plot of the PLS model^47^

We can verify the allotment of the molecules in the latent variable space defined by the scores using the score plot (Fig. 3B). In this work, we have plotted the scores of first two components t1 and t2. From this plot, we can easily identify the similar or dissimilar compounds with respect to the odorant property of odorant molecules present in coffee. Fig. 3B shows that compound no. 13 (dimethyl trisulfide), 15 (methional), 16 (3-mercapto-3-methylbutyl formate), 18 (2-methyl-3-furanthiol), 19 (3-methyl-2-butene-1-thiol) and 20 (methanethiol) are situated in the upper right hand corner bearing similar properties (all these compounds contain sulphur atom(s)) whereas the compounds which are far apart from each other like those in the lower left hand corner (24 (4-hydroxy-2,5-dimethyl-3(2*H*)-furanone) and 25 (5-ethyl-3-hydroxy-4-methyl-2-(5*H*)-furanone)) and upper right hand corner (18 (2-methyl-3-furanthiol) and 19 (3-methyl-2-butene-1-thiol)) represent dissimilar compounds. On the other hand, the compounds which are in the center of the plane bear average properties. We can also identify the outliers from this plot. The compounds, which are situated outside the ellipse are indicated as outliers. In this figure, we have found that compound no. 30 ((*E*)-β-damascenone) is situated outside the ellipse and indicated as an outlier (Fig. 3B).

#### 3.2.2 Loading plot of the PLS model^47^

From the loading plot (Fig. 3C), we have found that the descriptors, C-029 and B01[C–S] variables are directly correlated with the odorant property due their closeness to the *Y*-variable (log(1/OT)) while the descriptors H-049 and nHDon are inversely correlated with the odorant property of the molecules as these descriptors are situated in the opposite side of the *Y*-variable. Thus, B01[C–S] and C-029 descriptors are influential to the odorant property of the compounds as shown in compound no. 13 (dimethyl trisulfide), 16 (3-mercapto-3-methylbutyl formate), 18 (2-methyl-3-furanthiol), 19 (3-methyl-2-butene-1-thiol), 40 (2-methoxy-3,5-dimethylpyrazine) and 41 (2-methoxy-3-isopropylpyrazine) while H-049 and nHDon descriptors are detrimental towards the odorant property as shown in compound no. 22 (2-ethyl-4-hydroxy-5-methyl-3(2*H*)-furanone), 32 (4-ethyl guaiacol), 34 (vanillin), 35 (2,3-dimethylpyrazine), 42 (2-methoxy-3-isopropylpyrazine) and 43 (pyridine). The loading plot also showed that all the *X*-variables are loaded strongly in the response and divided into two groups. The first group is formed by B01[C–S], C-029, nHDon and H-049 descriptors while the second group is formed by F05[C–C] and ETA_Eta descriptors which have positive impact towards the odorant property but these descriptors are not similar to the other four descriptors.

### 3.3 Applicability domain

We have checked the applicability domain (99% confidence level) of the developed PLS models. The PLS model developed from the odorants present in black tea (Fig. S7†) showed that all the test set compounds are within the critical DModX value (*D*-critical = 3.553). On the other hand, the PLS model developed from the odorants present in coffee (Fig. S8†) showed that all the test set compounds are within the applicability domain (*D*-critical = 2.626).

## 4. Conclusion

PLS regression-based modeling technique was employed separately using odorant property (log(1/OT)) of diverse classes of constituents present in black tea and coffee to find out the key structural attributes of the components which make these beverages attractive worldwide. We have also investigated the key structural properties which make the odor difference between tea and coffee using this *in silico* approach. Prior to development of the final models, we have used a variable selection approach which proved to be an efficient strategy to extract the significant descriptors for development of final models. The statistical results obtained from various validation strategies justify the reliability and usefulness of the developed predictive PLS models. The PLS models were developed keeping in mind the OECD principles for QSPR model development. From the insights obtained from the developed PLS models, we found out that relative hydrophobic surface area, molecules having heteroatoms with higher number of multiple bonds, molecules containing hydrogen atom connected with C^3^(sp^3^)/C^2^(sp^2^)/C^3^(sp^2^)/C^3^(sp) fragments, electron-richness, C–O atom pairs at topological distance 10 and surface weighted charged partial negative surface areas are the key properties which regulate the odorant properties of black tea. On the other hand, C–S atom pairs at topological distance 1, C–C atom pair at topological distance five, donor atoms like N and O for hydrogen bonds, hydrogen atom connected with C^3^(sp^3^)/C^2^(sp^2^)/C^3^(sp^2^)/C^3^(sp) fragments and R–CX–X fragments (where, R represents any group linked through carbon and X represents any heteroatom (O, N, S, P, Se, and halogens)) play crucial roles to regulate the odorant properties of coffee. It was obviously difficult to compare the two beverages because they share little common molecules (for example, a large number of unsaturated aldehydes are present in tea while there is only one in coffee); consequently there is an understandable lack of common descriptors (except H-049 that has not the same importance range in the models). However, this is interesting to note that the influence of C^3^(sp^3^)/C^2^(sp^2^)/C^3^(sp^2^)/C^3^(sp) fragments to regulate the odorant properties are opposite in the constituents present in black tea and coffee. Thus, it can be concluded that to enhance the odorant properties of the constituents present in black tea, (i) the numerical value of relative hydrophobic surface area (Jurs-RASA) of the molecules should be more than 0.757; (ii) molecules should contain hydrogen atom connected with C^3^(sp^3^)/C^2^(sp^2^)/C^3^(sp^2^)/C^3^(sp) fragments; (iii) molecules should contain heteroatoms with higher number of multiple bonds; (iv) molecule should be less electron-rich; (v) molecules should not contain C–O atom pairs at topological distance 10; and (vi) the numerical value of surface weighted charged partial negative surface areas of the molecules should be in higher range. On the other hand, to enhance the odorant properties of the constituents present in coffee, (i) the molecules should contain C–S atom pairs at topological distance 1; (ii) the molecules should contain C–C atom pairs at topological distance five; (iii) molecules should not contain donor atoms like N and O for hydrogen bonds; (iv) molecules should not contain any hydrogen atom connected with C^3^(sp^3^)/C^2^(sp^2^)/C^3^(sp^2^)/C^3^(sp) fragments; and (v) the molecules should contain R–CX–X fragments (where, R represents any group linked through carbon and X represents any heteroatom (O, N, S, P, Se, and halogens)). Thus, the developed models can be successfully utilized for *in silico* prediction of odorant properties of diverse classes of compounds if they fall within the AD of the developed PLS models and also give us the key information which makes the odor difference between tea and coffee.

## Conflicts of interest

There are no conflicts to declare.

## Supplementary Material

RA-008-C7RA12914A-s001

## References

[cit1] Alasalvar C., Topal B., Serpen A., Bahar B., Pelvan E., Gökmen V. (2012). Flavor characteristics of seven grades of black tea produced in Turkey. J. Agric. Food Chem..

[cit2] FAO , FAOSTAT-Tea Production, FAO, Rome, Italy, 2009

[cit3] Yamanishi T. and Kobayashi A., Progress of tea aroma chemistry, Kluwer Academic/Plenum Publishers, New York, 1999, pp. 135–145

[cit4] Borse B. B., Rao L. J. M., Nagalakshmi S., Krishnamurthy N. (2002). Fingerprint of black teas from India: identification of the regio-specific characteristics. Food Chem..

[cit5] Bhattacharyya N., Bandyopadhyay R., Bhuyan M., Tudu B., Ghosh D., Jana A. (2008). Electronic nose for black tea classification and correlation of measurements with “Tea Taster” marks. IEEE Trans. Instrum. Meas..

[cit6] Rawat R., Gulati A., Babu G. K., Acharya R., Kaul V. K., Singh B. (2007). Characterization of volatile components of Kangra orthodox black tea by gas chromatography-mass spectrometry. Food Chem..

[cit7] Yeretzian C. , Hand book of Odor, ed. A. Buettner, Springer, 2017, ch. 6, pp. 107–128

[cit8] https://en.wikipedia.org/wiki/List_of_countries_by_coffee_production

[cit9] Leonardos G., Kendall D., Barnard N. (1969). Odor Threshold Determinations of 53 Odorant Chemicals. J. Air Pollut. Control Assoc..

[cit10] Tierney K. B., Ross P. S., Jarrard H. E., Delaney K. R., Kennedy C. J. (2006). Changes in juvenile coho salmon electro-olfactogram during and after short-term exposure to current-use pesticides. Environ. Toxicol. Chem..

[cit11] Paibon W., Yimnoi C. A., Tembab N., Boonlue W., Jampachaisri K., Nuengchamnong N., Waranuch N., Ingkaninan K. (2011). Comparison and evaluation of volatile oils from three different extraction methods for some Thai fragrant flowers. Int. J. Cosmet. Sci..

[cit12] Cometto-Muñiz J. E., Cain W. S., Abraham M. H., Gil-Lostes J. (2008). Concentration-detection functions for the odor of homologous n-acetate esters. Physiol. Behav..

[cit13] Rappert S., Müller R. (2005). Odor compounds in waste gas emissions from agricultural operations and food industries. Waste Manage..

[cit14] Benzo M., Gilardoni G., Gandini C., Caccialanza G., Finzi P. V., Vidari G., Abdod S., Layedra P. (2007). Determination of the threshold odor concentration of main odorants in essential oils using gas chromatography–olfactometry incremental dilution technique. J. Chromatogr. A.

[cit15] Espino-Díaz M., Sepúlveda D. R., González-Aguilar G., Olivas G. I. (2016). Biochemistry of Apple Aroma: A Review. Food Sci. Biotechnol..

[cit16] Ojha P. K., Roy K. (2017). Development of a robust and validated 2D-QSPR model for sweetness potency of diverse functional organic molecules. Food Chem. Toxicol..

[cit17] Das S., Ojha P. K., Roy K. (2017). Multilayered variable selection in QSPR: a case study of modeling melting point of bromide ionic liquids. International Journal Quantitative Structure-Property Relationship.

[cit18] Toropov A. A., Toropova A. P., Cappellini L., Benfenati E., Davoli E. (2016). Odor threshold prediction by means of the Monte Carlo method. Ecotoxicol. Environ. Saf..

[cit19] http://www.oecd.org/dataoecd/33/37/37849783.pdf

[cit20] Magagna F., Cordero C., Cagliero C., Liberto E., Rubiolo P., Sgorbini B., Bicchi C. (2017). Black tea volatiles fingerprinting by comprehensive two-dimensional gas chromatography–Mass spectrometry combined with high concentration capacity sample preparation techniques: Toward a fully automated sensomic assessment. Food Chem..

[cit21] Talete , Dragon (Version 6) Srl, 2010, Retrieved from. http://www.talete.mi.it/products/dragondescription.htm

[cit22] Cerius2 Version 4.10 is a product of Accelrys Inc., San Diego, CA, USA, 2005, accessed from http://www.accelrys.com

[cit23] Roy P. P., Leonard J. T., Roy K. (2008). Exploring the impact of the size of training sets for the development of predictive QSAR models. Chemom. Intell. Lab. Syst..

[cit24] Park H. S., Jun C. H. (2009). A simple and fast algorithm for K-medoids clustering. Expert Systems with Applications.

[cit25] Rogers D., Hopfinger A. J. (1994). Application of genetic function approximation to quantitative structure-activity relationships and quantitative structure-property relationships. J. Chem. Inf. Comput. Sci..

[cit26] Roy K., Das R. N., Ambure P., Aher R. B. (2016). Be aware of error measures. Further studies on validation of predictive QSAR models. Chemom. Intell. Lab. Syst..

[cit27] Darlington R. B. , in Regression and linear models, McGraw- Hill, New York, 1990

[cit28] Wold S., Sjöström M., Eriksson L. (2001). PLS-regression: a basic tool of chemometrics. Chemom. Intell. Lab. Syst..

[cit29] Holland J. H. , Adaptation in natural and artificial systems. An introductory analysis with application to biology, control, and artificial intelligence, University of Michigan Press, Ann Arbor, MI, 1975

[cit30] Hopfinger A. J. , Koehler M. G. and Rogers D., Molecular modeling of polymers, 14 quantitative structure-property relationship analyses of multicomponent systems containing polymers, in Macromolecular Symposia, Hüthig&WepfVerlag, 1995, vol. 98, pp. 1087–1100

[cit31] Fan Y., Shi L. M., Kohn K. W., Pommier Y., Weinstein J. N. (2001). Quantitative structure antitumor activity relationships of camptothecin analogues: cluster analysis and genetic algorithm-based studies. J. Med. Chem..

[cit32] Snedecor G. W. and Cochran W. G., Statistical Methods, Oxford & IBH, New Delhi, 1967

[cit33] Hawkins D. M., Basak S. C., Mills D. (2003). Assessing model fit by cross-validation. J. Chem. Inf. Comput. Sci..

[cit34] Schüürmann G., Ebert R. U., Chen J., Wang B., Kuhne R. (2008). External validation and prediction employing the predictive squared correlation coefficients test set activity mean *vs.* training set activity mean. J. Chem. Inf. Model..

[cit35] Hawkins D. M. (2004). The problem of overfitting. J. Chem. Inf. Comput. Sci..

[cit36] Chirico N., Gramatica P. (2011). Real external predictivity of QSAR models: how to evaluate it? Comparison of different validation criteria and proposal of using the concordance correlation coefficient. J. Chem. Inf. Model..

[cit37] Lawrence I., Lin K. (1992). Assay validation using the concordance correlation coefficient. Biometrics.

[cit38] Ojha P. K., Mitra I., Das R. N., Roy K. (2011). Further exploring *r*_m_^2^ metrics for validation of QSPR models. Chemom. Intell. Lab. Syst..

[cit39] Roy K., Chakraborty P., Mitra I., Ojha P. K., Kar S., Das R. N. (2013). Some case studies on application of “*r*_m_^2^” metrics for judging quality of quantitative structure–activity relationship predictions: emphasis on scaling of response data. J. Comput. Chem..

[cit40] Roy K., Mitra I., Kar S., Ojha P. K., Das R. N., Kabir H. (2012). Comparative studies on some metrics for external validation of QSPR models. J. Chem. Inf. Model..

[cit41] Melagraki G., Afantitis A. (2013). Exploring corrosion inhibition of steel in acidic medium. Chemom. Intell. Lab. Syst..

[cit42] UMETRICS , UMETRICS SIMCA-P 10.0, info@umetrics.com, www.umetrics.com, Umea, Sweden, 2002

[cit43] Golbraikh A., Tropsha A. (2002). Beware of q^2^!. J. Mol. Graphics Modell..

[cit44] MINITAB is a statistical software of Minitab Inc., USA, http://www.minitab.com/en-US/default.aspx

[cit45] Johnson S. R. (2008). The trouble with QSAR (or how I learned to stop worrying and embrace fallacy). J. Chem. Inf. Model..

[cit46] Farrés M., Platikanov S., Tsakovski S., Tauler R. (2015). Comparison of the variable importance in projection (VIP) and of the selectivity ratio (SR) methods for variable selection and interpretation. J. Chemom..

[cit47] Eriksson L., Jaworska J., Worth A. P., Cronin M. T., McDowell R. M., Gramatica P. (2003). Methods for reliability and uncertainty assessment and for applicability evaluations of classification-and regression-based QSARs. Environ. Health Perspect..

[cit48] Jackson J. E. , A Users Guide to Principal Components, John Wiley &Sons Inc., Canada, 2005, vol. 587

