# Supplementary material for: PLS regression-based chemometric modeling of odorant properties of diverse chemical constituents of black tea and coffee
Source: RSC Adv. 2018 Jan 9;8(5):2293–304. doi: 10.1039/c7ra12914a (PMC9092630; doi:10.1039/c7ra12914a)
Supplement: RA-008-C7RA12914A-s001 [file RA-008-C7RA12914A-s001.pdf]

Electronic Supplementary Material (ESI) for RSC Advances.
This journal is © The Royal Society of Chemistry 2017

PLS regression-based chemometric modeling of odorant property of diverse chemical constituents of black tea and coffee

Probir Kumar Ojha and Kunal Roy*

Drug Theoretics and Cheminformatics Laboratory,

Department of Pharmaceutical Technology,

Jadavpur University, Kolkata 700 032, India,

Email: kunalroy_in@yahoo.com; mailto:kunal.roy@jadavpuruniversity.in

Phone: +91 98315 94140; Fax: +91-33-2837-1078;

URL: http://sites.google.com/site/kunalroyindia/

Supplementary Materials

Table S1 List of aroma components present in black tea with observed and model derived log(1/OT) values

Compound no.	SMILES notation	Chemical structure	Structure Name	Chemical class	Observed value (log(1/OT))	Predicted value
1	CC(C)C=O	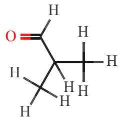	2-Methyl propanal	Aldehyde	4.50	2.77
2	CC(=O)C	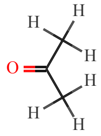	Acetone	Ketone	-0.93	0.72
3	CCCC=O	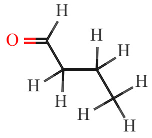	Butanal	Aldehyde	3.60	2.64
4	CCOC(=O)C	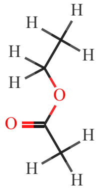	Ethyl acetate	Ester	1.97	2.33
5	CCCCO	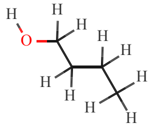	1-Butanol	Alcohol	2.69	2.25
6	CC(C)CC=O	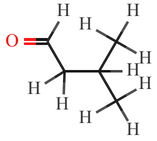	3-Methyl butanal	Aldehyde	3.82	3.39

7	CC[C@@H](C)C=O	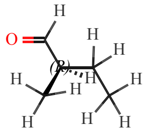	2-Methyl butanal	Aldehyde	3.94	3.55
8	CC(=O)O	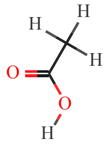	Acetic acid	Acid	0.08	0.56
9	CC[C@H](O)C=C	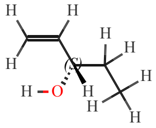	1-Penten-3-ol	Alcohol	2.33	2.53
10	CCCCC=O	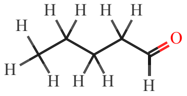	Pentanal	Aldehyde	3.33	3.22
11	CCC(=O)O	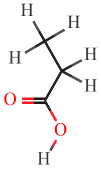	Propanoic acid	Acid	0.57	1.17
12	CC(C)C(=O)O	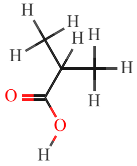	2-Methyl propanoic acid	Acid	1.04	1.73
13	CC/C=C/C=O	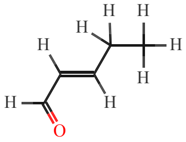	(E)-2-Pentenal	Aldehyde	2.45	2.37
14*	CCCCCO	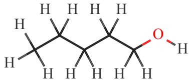	1-Pentanol	Alcohol	1.47	2.49

15	CCCC(=O)O	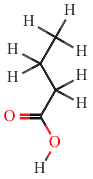	Butanoic acid	Acid	2.56	1.52
16*	CCCCCC=O	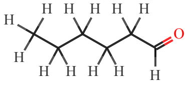	Hexanal	Aldehyde	3.10	3.61
17	O=Cc1ccco1	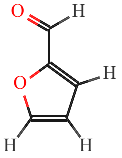	Furfural	Aldehyde	1.51	0.71
18	CC(C)CC(=O)O	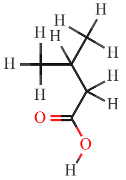	3-Methyl butanoic acid	Acid	2.16	1.97
19	CC[C@@H](C)C(=O)O	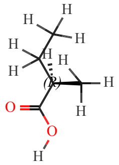	2-Methyl butanoic acid	Acid	2.16	2.09
20*	CCC/C=C/C=O	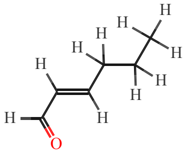	(E)-2-Hexenal	Aldehyde	2.37	3.12
21	CC/C=C\CCO	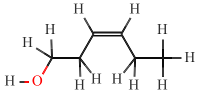	(Z)-3-hexen-1-ol	Alcohol	1.82	2.53

22	CCC/C=C/CO	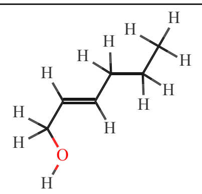	(E)-2-hexen-1-ol	Alcohol	1.30	2.51
23*	CCCCCCO	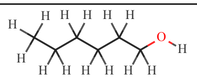	1-Hexanol	Alcohol	2.41	2.61
24*	CCCCC(=O)O	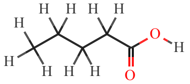	Pentanoic acid	Acid	1.53	1.75
25	CCCCCC(=O)C	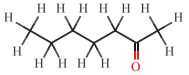	2-Heptanone	Ketone	2.58	2.90
26	CC/C=C\CCC=O	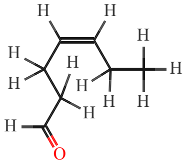	(Z)-4-heptenal	Aldehyde	6.27	3.89
27	CCCCCCC=O	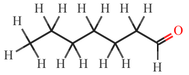	Heptanal	Aldehyde	2.36	3.87
28*	C(=O)/C=C/C=C/C	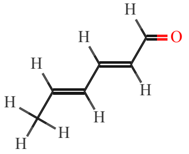	(E,E)-2,4-Hexadienal	Aldehyde	3.98	2.52
29	CCCC/C=C/C=O	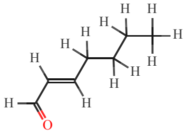	(E)-2-Heptenal	Aldehyde	4.35	3.65
30	O=Cc1ccccc1	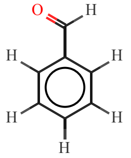	Benzaldehyde	Aldehyde	2.48	2.28

31	CCCCCCCO	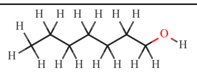	1-Heptanol	Alcohol	4.59	2.83
32	CCCCC[C@@H](O) C=C	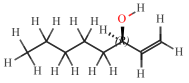	1-Octen-3-ol	Alcohol	3.41	3.74
33	CC(=CCCC(=O)C)C	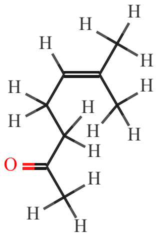	6-Methyl-5-hepten-2-one	Ketone	2.10	3.34
34	CCCCCCC(=O)C	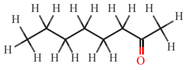	2-Octanone	Ketone	2.40	3.43
35*	CCCCCC(=O)O	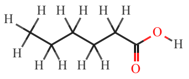	Hexanoic acid	Acid	1.59	1.89
36*	CCCCC[C@@H]1CC CO1	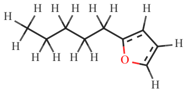	2-Pentyl furan	Furan	4.36	3.91
37	CC/C=C\CCOC(=O)C	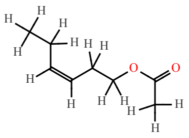	(Z)-3-Hexen-1-ol acetate	Ester	4.25	4.05
38*	CCCCCCCC=O	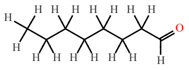	Octanal	Aldehyde	2.60	4.14
39	C(=C\C=O)/C=C/CC	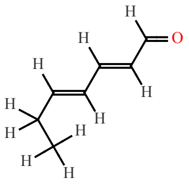	(E,E) 2,4-Heptadienal	Aldehyde	2.49	3.43

40*	CC(=C)[C@H]1CCC(=CC1)C	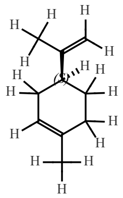	Limonene	Terpene	4.13	5.30
41	OCc1ccccc1	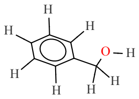	Benzyl alcohol	Alcohol	1.03	1.66
42*	O=CCc1ccccc1	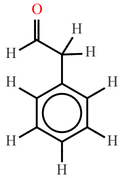	Phenyl acetaldehyde	Aldehyde	4.28	3.52
43	CCCCC/C=C/C=O	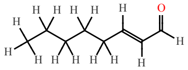	(E)-2-Octenal	Aldehyde	4.50	4.08
44	CC(=O)/C=C/C=C/CC	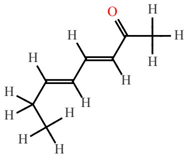	(E,E)-3,5-Octadien-2-one	Ketone	5.40	3.36
45	CCCCCCCCO	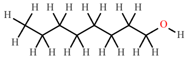	1-Octanol	Alcohol	3.07	2.94
46*	CCCCCCC(=O)O	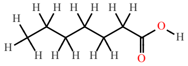	Heptanoic acid	Acid	1.64	2.03
47	CC(=CCC[C@](C)(O)C=C)C	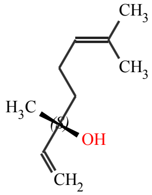	Linalool	Alcohol	5.41	5.21

48	CC(=C/C=C/C(=O)C)C	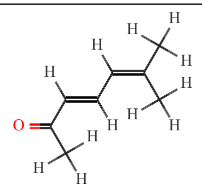	6-Methyl-3,5-heptadien-2-one	Ketone	2.51	3.44
49	CCCCCCCCC=O	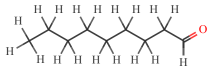	Nonanal	Aldehyde	2.98	4.32
50	OCCc1ccccc1	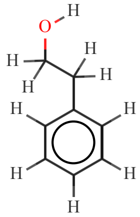	2-Phenyl ethanol	Alcohol	2.09	2.22
51	CC/C=C\CC/C=C/C=O	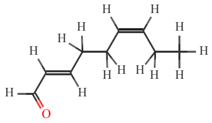	(E,Z)-2,6-Nonadienal	Aldehyde	7.66	4.65
52	CCCCCC/C=C/C=O	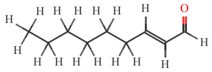	(E)-2-Nonenal	Aldehyde	2.19	4.40
53*	CCCCCCCCCO	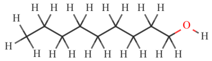	1-Nonanol	Alcohol	3.46	2.95
54	CCCCCCCC(=O)O	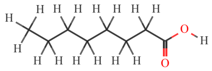	Octanoic acid	Acid	1.68	1.96
55	CCCCCCCCCC=O	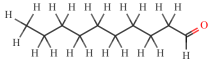	Decanal	Aldehyde	2.38	3.95
56	CCCC/C=C/C=C/C=O	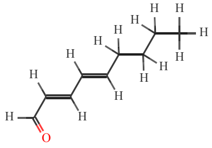	(E,E)-2,4-Nonadienal	Aldehyde	5.94	4.82

57*	CC1=C(C=O)C(C)(C)CCC1	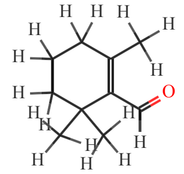	β-Cyclocitral	Aldehyde	4.48	5.99
58	CC(=CCC/C(=C/CO)/C)C	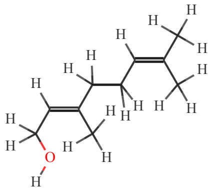	Geraniol	Terpenol	4.68	3.80
59*	CCCCCCC/C=C/C=O	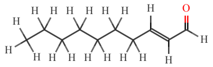	(E)-2-Decenal	Aldehyde	4.19	4.10
60	CC(=CCC/C(=C/C=O)/C)C	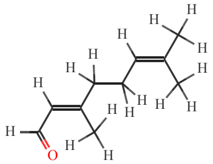	Geranial	Terpene	3.68	5.19
61	CCCCCCCCC(=O)O	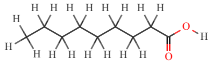	Nonanoic acid	Acid	1.72	2.11
62	CCCCCCCCCC(=O)C	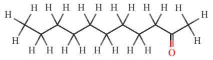	2-Undecanone	Ketone	4.39	2.95
63*	C(/C=C\C=C\C=O)CCCC	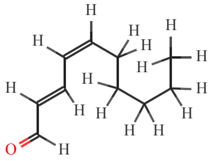	(E,Z)-2,4-Decadienal	Aldehyde	4.18	4.36
64*	CCCCCCCCCCC=O	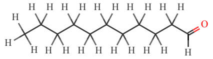	Undecanal	Aldehyde	4.53	4.00
65	CCCCC/C=C/C=C/C=O	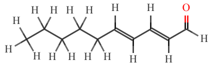	(E,E)-2,4-Decadienal	Aldehyde	2.93	4.33
66	CCCCC[C@@H]1CCC(=O)O1	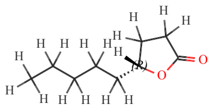	Dihydro-5-pentyl-2(3H)-Furanone	Furanone	3.72	3.22

67	CCCCCCCCCC(=O)O	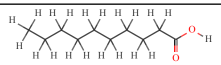	Decanoic acid	Acid	1.24	0.80
68	O=C(C1=C(C=CCC1(C)C)C)/C=C/C	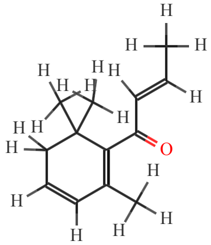	β-Damascenone	Norisoprenoid	7.68	7.09
69	COc1cc(C=O)ccc1O	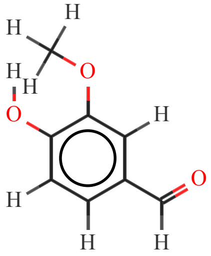	Vanillin	Phenolic	3.88	4.61
70	CCCCCCCCCCCC=O	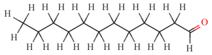	Dodecanal	Aldehyde	4.96	4.12
71	CC(=O)/C=C/[C@@H]1C(=CCCC1(C)C)C	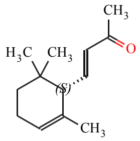	α-Ionone	Norisoprenoid	4.59	5.54
72	CC(=CCC/C(=C/CCC(=O)C)/C)C	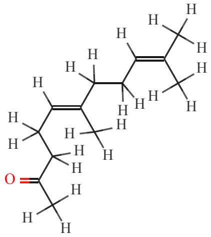	Geranyl acetone	terpene	3.51	3.79

73*	CC(=O)/C=C/C1=C(C)CCCC1(C)C	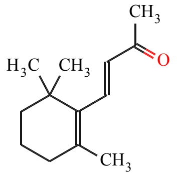	β-Ionone	Norisoprenoid	5.98	5.74
74	CCCCCCCCCCCC(=O)O	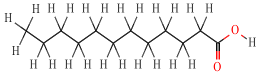	Dodecanoic acid	Acid	1.30	0.99
75*	CCCCCCCCCCCCCC=O	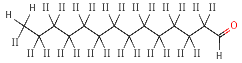	Tetradecanal	Aldehyde	3.55	4.17
76	CCCCCCCCCCCCCC(=O)O	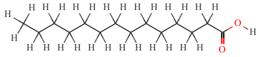	Tetradecanoic acid	Acid	1.36	1.18

*denotes test set compounds

Table S2 List of aroma components present in coffee with observed and model derived log(1/OT) values

Compound No.	Smiles notation	Chemical structure	Structure name	Chemical class	Observed value ((log(1/OT))	Predicted value
1*	CC[C@@H](C)C=O	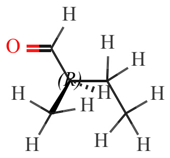	2-Methylbutanal	Aldehyde	1.82	0.92
2	CC(C)CC=O	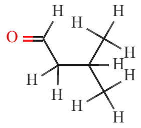	3-Methylbutanal	Aldehyde	2.39	0.92
3*	CCCCCC/C=C/C=O	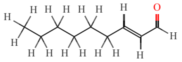	(E)-2-Nonenal	Aldehyde	3.24	2.38
4	CC=O	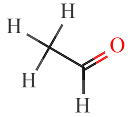	Acetaldehyde	Aldehyde	1.80	0.55
5	COc1ccc(C=O)cc1	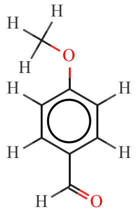	4-Methoxybenzaldehyde	Aldehyde	0.70	1.19

6	CCC=O	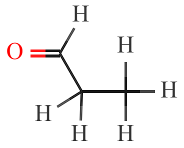	Propanal	Aldehyde	0.76	0.65
7	CC[C@@H](C)C(=O)O	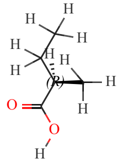	2-Methylbutyric acid	Acid	1.01	0.88
8	CC(C)CC(=O)O	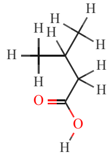	3-Methylbutyric acid	Acid	-0.84	0.88
9	CCCC[C@@H](C)C(=O)[O-]	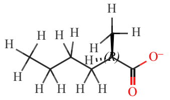	Ethyl 2-methylbutyrate	Ester	2.41	2.96
10	CCC[C@H](C)CC(=O)[O-]	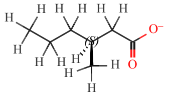	Ethyl 3-methylbutyrate	Ester	2.33	2.71
11*	O=Cc1ccco1	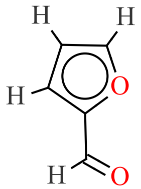	Furfural	Furan	-0.46	0.67
12	Cc1ccc(C=O)o1	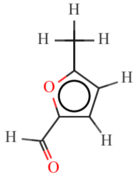	5-Methyl-2-furancarboxyaldehyde	Furan	-1.74	0.80

13	CSSSC	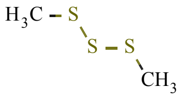	Dimethyl trisulfide	Sulfur containing compounds	4.10	4.55
14*	Cc1occc1SSc1ccoc1C	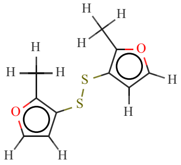	Bis(2-methyl-3-furyl)disulfide	Sulfur containing compounds	5.47	7.11
15	CSCCC=O	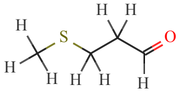	Methional	Sulfur containing compounds	2.72	3.28
16	CC(C)(S)CCOC=O	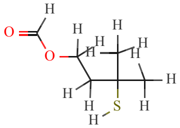	3-Mercapto-3-methylbutyl formate	Thiols	4.63	4.30
17*	SCc1ccco1	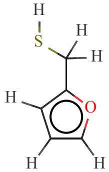	2-Furfurylthiol	Thiols	4.06	4.34
18	Cc1occc1S	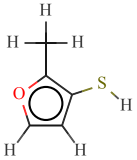	2-Methyl-3-furanthiol	Thiols	4.21	4.27

19	CC(=CCS)C	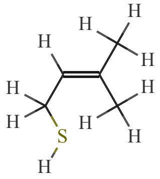	3-Methyl-2-butene-1-thiol	Thiols	5.53	4.49
20	CS	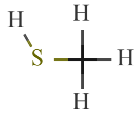	Methanethiol	Thiols	3.38	4.05
21	C[C@H]1OCCC1=O	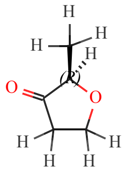	Dihydro-2-methyl-3(2H)-furanone	Furanone	4.30	2.05
22	CC[C@@H]1OC(=C(O)C1=O)C	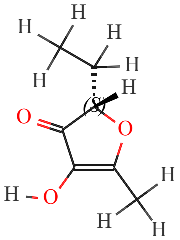	2-Ethyl-4-hydroxy-5-methyl-3(2H)-furanone	Furanone	0.85	1.40
23*	C[C@@H]1OC(=O)C(=C1C)O	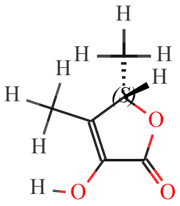	3-Hydroxy-4,5-dimethyl-2(5H)-furanone	Furanone	0.81	0.99

24	C[C@@H]1OC(=C(O)C1=O)C	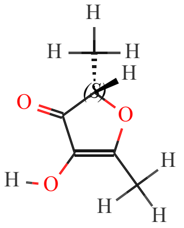	4-Hydroxy-2,5-dimethyl-3(2H)-furanone	Furanon<br>e	1.11	0.98
25	CC[C@@H]1OC(=O)C(=C1C)O	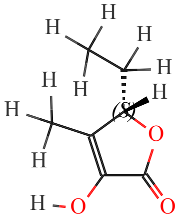	5-Ethyl-3-hydroxy-4-methyl-2(5H)-furanone	Furanon<br>e	1.28	1.15
26	CCC1=C(O)C(=O)[C@H](C)O1	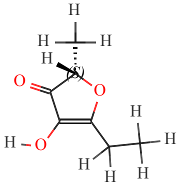	5-Ethyl-4-hydroxy-2-methyl-3(2H)-furanone	Furanon<br>e	2.09	1.40
27	CCCCCC(=O)C=C	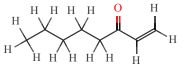	1-Octen-3-one	Ketone	4.55	3.08
28*	CC(=O)C(=O)C	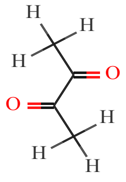	2,3-Butanedione	Ketone	2.46	1.88
29	CCC(=O)C(=O)C	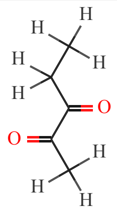	2,3-Pentanedione	Ketone	0.70	2.01

30	O=C(C1=C(C=CCC1(C)C)C)/C=C/C	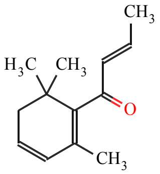	(E)-Beta-damascenone	Norisoprenoids	5.40	5.16
31	COc1ccccc1O	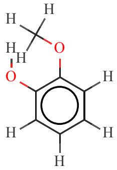	Guaiacol	Phenolic	1.70	0.99
32	CCc1ccc(O)c(OC)c1	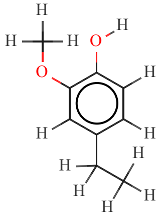	4-Ethyl Guaiacol	Phenolic	0.79	1.83
33	COc1cc(C=C)ccc1O	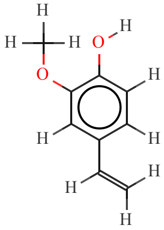	4-Vinyl Guaiacol	Phenolic	2.30	1.67
34	COc1cc(C=O)ccc1O	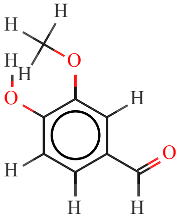	Vanillin	Phenolic	0.78	0.28

35	Cc1nccnc1C	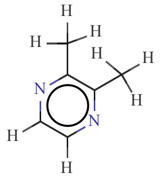	2,3-Dimethylpyrazine	Pyrazine	-0.87	-0.21
36*	Cc1cnc(C)cn1	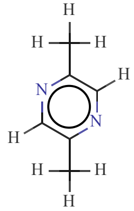	2,5-Dimethylpyrazine	Pyrazine	0.13	0.04
37	CCc1ncc(C)nc1CC	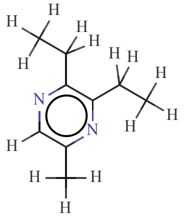	2,3-Diethyl-5-methylpyrazine	Pyrazine	3.22	2.68
38	CCc1ncc(C)nc1C	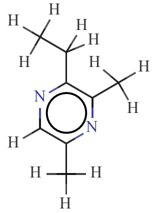	2-Ethyl-3,5-dimethylpyrazine	Pyrazine	3.53	1.73
39*	CCc1nc(C)cnc1C	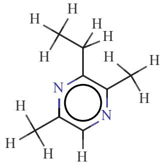	2-Ethyl-3,6-dimethyl-pyrazine	Pyrazine	1.20	2.00

40	COc1ncc(C)nc1C	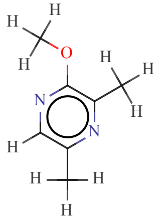	2-Methoxy-3,5-dimethylpyrazine	Pyrazine	4.36	4.54
41	COc1nccnc1C(C)C	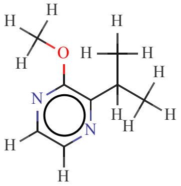	2-Methoxy-3-isopropylpyrazine	Pyrazine	4.88	4.65
42	CCc1cnccn1	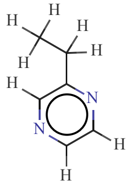	Ethylpyrazine	Pyrazine	-1.57	-1.09
43	c1ccncc1	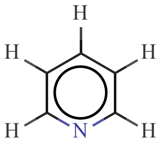	Pyridine	Pyridine	0.01	-0.47
44*	CC(=CCC[C@](C)(O)C=C)C	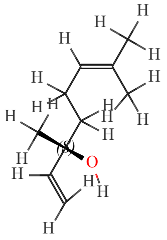	Linalool	Terpene	2.96	2.82

45	CC(=C)[C@H]1CCC(=CC1)C	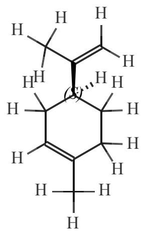	Limonene	Terpene	1.53	3.41
46	CC(=CCC/C(=C/CO)/C)C	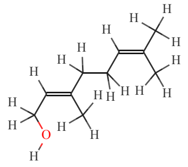	Geraniol	Terpene	2.15	2.78

*denotes test set compounds

Table S3 Different validation parameters employed to assess the quality and predictivity of the developed PLS models

**Type**	**Validation metrics used for regression**	**Explanation**	**References**
Goodness of fit	$R^2 = 1 - \frac{\sum (Y_{obs} - Y_{calc})^2}{\sum (Y_{obs} - \overline{Y}_{training})^2}$	Y_{obs}denotes the observed response values for the training set and Y_{calc} denotes the calculated response values for the training set of compounds. $\overline{Y}_{training}$is the mean observed response of the training set compounds.	32
	$R_a^2 = \frac{(n-1)R^2 - p}{n-p-1}$	n is the number of compounds and p is the number of descriptors	32
	$s = \sqrt{\frac{\sum (Y_{obs} - Y_{calc})^2}{n-p-1}}$	$Y_{obs}$and $Y_{calc}$are the actual and estimated scores respectively, while n is the number of compounds and p is the number of descriptors.	32
	$F = \frac{\frac{\sum (Y_{calc} - \overline{Y})^2}{p}}{\frac{\sum (Y_{obs} - Y_{calc})^2}{n-p-1}}$	The degrees of freedom of F is *p*, (*n-p-1*) where p is number of independent variables (in case of MLR) or number of components (in case of PLS) and n is the total number of data points. F value describes stability of the regression coefficients.	32
Robustness parameter	$Q^2 (or Q^2_{LOO}) = 1 - \frac{\sum (Y_{obs(training)} - Y_{pred(training)})^2}{\sum (Y_{obs(training)} - \overline{Y}_{(training)})^2}$	Cross-validated R^2 (Q^2) is checked for internal validation. $Y_{obs(training)}$ is the observed response, $Y_{pred(training)}$ is the predicted response of the training set molecules based on the leave-one-out (LOO) technique. The generally accepted threshold value of Q^2 is 0.5	33
Predictivityparameter/criteria	$Q^2_{F1} = 1 - \frac{\sum (Y_{obs(test)} - Y_{pred(test)})^2}{\sum (Y_{obs(test)} - \overline{Y}_{training})^2}$	The metric $Q^2_{(F1)}$ or R^2_{pred} is a measure of correlation between the observed and predicted data of test set. $Y_{obs(test)}$ and $Y_{pred(test)}$ signify the observed and predicted response data for the test set molecules,	34

		while $\overline{Y}_{training}$ denotes the mean observed response of the training set. The stipulated threshold value for $Q^2_{(F1)}$ is 0.5.	
	$Q^2_{F2} = 1 - \frac{\sum (Y_{obs(test)} - Y_{pred(test)})^2}{\sum (Y_{obs(test)} - \overline{Y}_{test})^2}$	In case of $Q^2_{(F2)}$, the $\overline{Y}_{test}$ term used in the denominator is the mean observed response of the test set. Almost equal or closer values of $Q^2_{(F2)}$ and $Q^2_{(F1)}$ infer that the training set mean lies in the close propinquity to that of the test set which indicate that the test set utilized for modeling covers the whole response domain of the model. The stipulated threshold value for $Q^2_{(F2)}$ is 0.5	35
Other metrics (defining both robustness and external predictive power)	$CCC = \overline{\rho}_c = \frac{2\sum_{i=1}^{n}(x_i - \overline{x})(y_i - \overline{y})}{\sum_{i=1}^{n}(x_i - \overline{x})^2 + \sum_{i=1}^{n}(y_i - \overline{y})^2 + n(\overline{x} - \overline{y})}$	The Concordance Correlation Coefficient measures both precision and accuracy detecting the distance of the observations from the fitting line and the degree of deviation of the regression line from that passing through the origin respectively. Any deviation of the regression line from the concordance line give a value of CCC smaller than 1. x_i and y_i refer to the observed and predicted response values of the test set, n denotes the number of compounds, and $\overline{x_i}$ and $\overline{y_i}$ signify the mean of observed and predicted values, respectively.	36-37
	$\overline{r_m^2} = \frac{(r_m^2 + r'^2_m)}{2}$ and , $\Delta r_m^2 = \left\| r_m^2 - r'^2_m \right\|$ where, $r_m^2 = r^2 \times \left(1 - \sqrt{r^2 - r_0^2}\right)$ $r'^2_m = r^2 \times \left(1 - \sqrt{r^2 - r'^2_0}\right)$ The parameters r^2 and r_0^2 are defined as	The r_m^2 metrics for internal validation [$\overline{r_m^2}_{(LOO)}$ and $\Delta r_m^2{}_{(LOO)}$] reflect model predictability in a better way. Similarly, in order to find out the propinquity between the observed and predicted activity data for test set of compounds, the metrics $r_m^2{}_{(test)}$[$\overline{r_m^2}_{(test)}$, $\Delta r_m^2{}_{(test)}$] were also introduced. The metrics, $\overline{r_m^2}_{(overall)}$ and $\Delta r_m^2{}_{(overall)}$ reflect the overall	37-40

	follows: $$r_0^2 = 1 - \frac{\sum (Y_{obs} - k \times Y_{pred})^2}{\sum (Y_{obs} - \overline{Y}_{obs})^2}$$ $$\& \, r_0'^2 = 1 - \frac{\sum (Y_{pred} - k' \times Y_{obs})^2}{\sum (Y_{pred} - \overline{Y}_{pred})^2}$$ The terms *k* and *k'* are defined as: $$k = \frac{\sum (Y_{obs} \times Y_{pred})}{\sum (Y_{pred})^2} \; \& \; k' = \frac{\sum (Y_{obs} \times Y_{pred})}{\sum (Y_{obs})^2}$$	predictability of the model for the entire dataset. For the acceptable prediction, the value of all Δr_m^2metrics should preferably be lower than 0.2 provided that the value of $\overline{r_m^2}$ is more than 0.5.	
	$$MAE = \frac{\sum \lvert Y_{obs} - Y_{pred} \rvert}{n}$$	This is also known as average absolute error (AAE)). This can be considered to be a better index of errors in the context of predictive modeling studies. Because of the involvement of squared term of the prediction errors in the expression of RMSE, the variance of errors may be influenced for a set of data. That is, squaring the higher prediction error values will have more weight than the lower errors in the formalism of root mean square error (RMSE), while MAE provides an equal weight to all the errors. Thus, MAE is considered to be a simpler and more straight-forward determinant of prediction errors	26

Table S4. Descriptors values of the PLS model obtained from the constituents present in black tea.

No.	H-049	ETA_Eta_F	ETA_BetaP_ns	Jurs-WNSA-3	F10[C-O]	<Jurs-RASA-0.767154>
1	1	1.57338	0.3	-3.90148	0	0.035678633
2	0	1.41169	0.375	-3.0199883	0	0.02208025
3	1	1.45051	0.3	-3.9723736	0	0.034711113
4	0	2.42819	0.25	-3.7610585	0	0.029450151
5	0	0.66929	0	-3.3567823	0	0.008099873
6	1	1.65303	0.25	-4.1197726	0	0.062890628
7	1	1.71956	0.25	-3.9180997	0	0.069427509
8	0	1.80253	0.375	-4.6836399	0	0
9	0	1.93492	0.16667	-3.7571781	0	0.038936055
10	1	1.57126	0.25	-4.3940624	0	0.057331698
11	0	2.02107	0.3	-5.014211	0	0
12	0	2.3495	0.25	-5.21119	0	0
13	1	2.61308	0.5	-4.6142263	0	0.044758122
14	0	0.72292	0	-3.6316973	0	0.031446742
15	0	2.20285	0.25	-5.7137594	0	0
16	1	1.67333	0.21429	-4.9876407	0	0.074776221
17	1	4.64954	0.92857	-6.1495276	0	0
18	0	2.45246	0.21429	-5.6735465	0	0
19	0	2.53128	0.21429	-5.3533495	0	0
20	1	2.80332	0.42857	-5.0493259	0	0.065016157
21	0	1.82004	0.14286	-4.3915772	0	0.043804438
22	0	1.79248	0.14286	-4.5054019	0	0.046343117
23	0	0.76842	0	-4.4166009	0	0.052889671
24	0	2.35347	0.21429	-6.4973932	0	0
25	0	1.88944	0.1875	-4.6058433	0	0.106536843
26	1	2.83287	0.3125	-5.9057132	0	0.08052216
27	1	1.76151	0.1875	-5.5200419	0	0.085298405
28	1	3.87402	0.64286	-5.5017102	0	0.0560347
29	1	2.96406	0.375	-5.6869836	0	0.080739108
30	1	5.45097	0.9375	-4.7191738	0	0.070161385
31	0	0.80783	0	-4.4785054	0	0.071624042

32	0	2.30317	0.11111	-4.7798824	0	0.109835813
33	0	3.10296	0.27778	-5.2095812	0	0.113522301
34	0	1.96729	0.16667	-4.4153251	0	0.136255245
35	0	2.48126	0.1875	-7.325941	0	0
36	0	4.01696	0.5	-2.6511929	0	0.189063057
37	0	4.1316	0.25	-5.9473484	0	0.091210267
38	1	1.83906	0.16667	-5.9608178	0	0.09974869
39	1	4.13715	0.5625	-5.7432222	0	0.077306139
40	0	2.64767	0.2	-1.2399666	0	0.232846
41	0	4.74389	0.75	-2.8875006	0	0.065004198
42	1	5.87317	0.83333	-4.6597127	0	0.091249695
43	1	3.10265	0.33333	-6.0799897	0	0.091943618
44	0	4.65796	0.5	-5.1683824	0	0.121438181
45	0	0.84257	0	-4.7257904	0	0.083873669
46	0	2.592	0.16667	-7.8171152	0	0
47	0	3.97354	0.18182	-4.6767001	0	0.156544265
48	0	4.71226	0.5	-4.9755405	0	0.121582878
49	1	1.90823	0.15	-6.4934822	0	0.109256037
50	0	5.05824	0.66667	-4.0558914	0	0.060429342
51	1	4.36108	0.4	-7.0242075	0	0.099237537
52	1	3.22434	0.3	-6.7245066	0	0.103724198
53	0	0.87362	0	-5.4150197	0	0.093253085
54	0	2.68962	0.15	-9.3987672	0	0
55	1	1.97063	0.13636	-6.7058102	1	0.122260622
56	1	4.55816	0.45	-5.9085796	0	0.112279691
57	1	3.85311	0.27273	-3.6838664	0	0.141597072
58	0	3.34144	0.18182	-5.9535794	0	0.090881424
59	1	3.3328	0.27273	-7.1835019	1	0.114546326
60	1	4.66116	0.36364	-7.1515107	0	0.106138765
61	0	2.77686	0.13636	-9.4831346	0	0
62	0	2.15235	0.125	-6.4945652	1	0.140503553
63	1	4.72765	0.40909	-7.4701979	1	0.111314103
64	1	2.02747	0.125	-7.5643321	1	0.128752822
65	1	4.72765	0.40909	-7.615695	1	0.111502664
66	0	2.95106	0.13636	-7.0392527	0	0.055970731
67	0	2.85569	0.125	-10.975581	2	0
68	0	7.95035	0.42857	-4.7179309	0	0.160453254
69	1	8.86796	0.77273	-10.36408	0	0
70	1	2.07965	0.11538	-7.8269952	1	0.134677298

71	0	5.77002	0.28571	-6.3553449	0	0.128937769
72	0	4.96007	0.25	-7.8380099	2	0.144804661
73	0	5.97579	0.32143	-5.6555548	0	0.142427946
74	0	2.99365	0.10714	-12.741923	2	0.0241023
75	1	2.17268	0.1	-9.3845361	1	0.147330742
76	0	3.11161	0.09375	-14.478269	2	0.053301616

Table S5 Descriptors values of the PLS model obtained from the constituents present in coffee.

No.	C-029	H-049	F05[C-C]	nHDon	B01[C-S]	ETA_Eta
1	0	1	0	0	0	4.01102
2	0	1	0	0	0	4.04565
3	0	1	4	0	0	7.60183
4	0	1	0	0	0	0.78298
5	0	1	1	0	0	4.06817
6	0	1	0	0	0	1.65033
7	0	0	0	1	0	5.02567
8	0	0	0	1	0	5.0241
9	0	0	2	0	0	7.69243
10	0	0	1	0	0	7.74386
11	0	1	0	0	0	1.88752
12	0	1	0	0	0	3.01612
13	0	0	0	0	1	5.68051
14	0	0	8	0	1	9.68117
15	0	1	0	0	1	4.21474
16	0	1	2	0	1	8.56811
17	0	0	0	0	1	3.79491
18	0	0	0	0	1	3.21863
19	0	0	0	0	1	5.17467
20	0	0	0	0	1	1.29099
21	0	0	0	0	0	4.29821
22	0	0	1	1	0	7.2908
23	0	0	0	1	0	5.99421
24	0	0	0	1	0	5.92212
25	0	0	0	1	0	7.37946
26	0	0	1	1	0	7.29023
27	0	0	3	0	0	6.39544

28	0	0	0	0	0	2.8077
29	0	0	0	0	0	3.9523
30	0	0	8	0	0	13.1398
31	0	0	1	1	0	3.69436
32	0	0	3	1	0	6.44191
33	0	0	3	1	0	5.05188
34	0	1	2	1	0	4.8424
35	0	2	0	0	0	3.78194
36	0	2	1	0	0	3.66167
37	0	1	5	0	0	8.00401
38	0	1	2	0	0	6.57169
39	0	1	3	0	0	6.58968
40	1	1	1	0	0	5.49247
41	1	2	5	0	0	6.96545
42	0	3	1	0	0	3.51662
43	0	2	0	0	0	1.52998
44	0	0	5	1	0	10.541
45	0	0	3	0	0	9.30522
46	0	0	5	1	0	10.1927

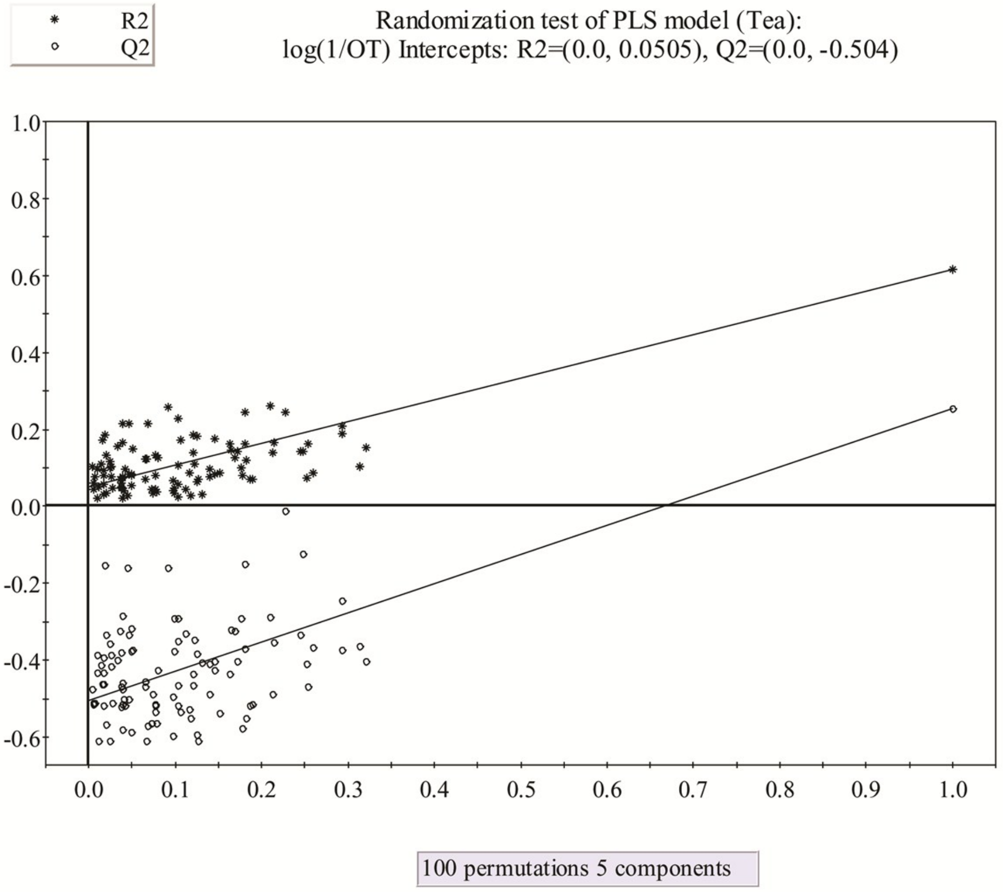

Fig. S1. The randomization plot for the QSPR model derived from PLS analysis using the odorants present in black tea.

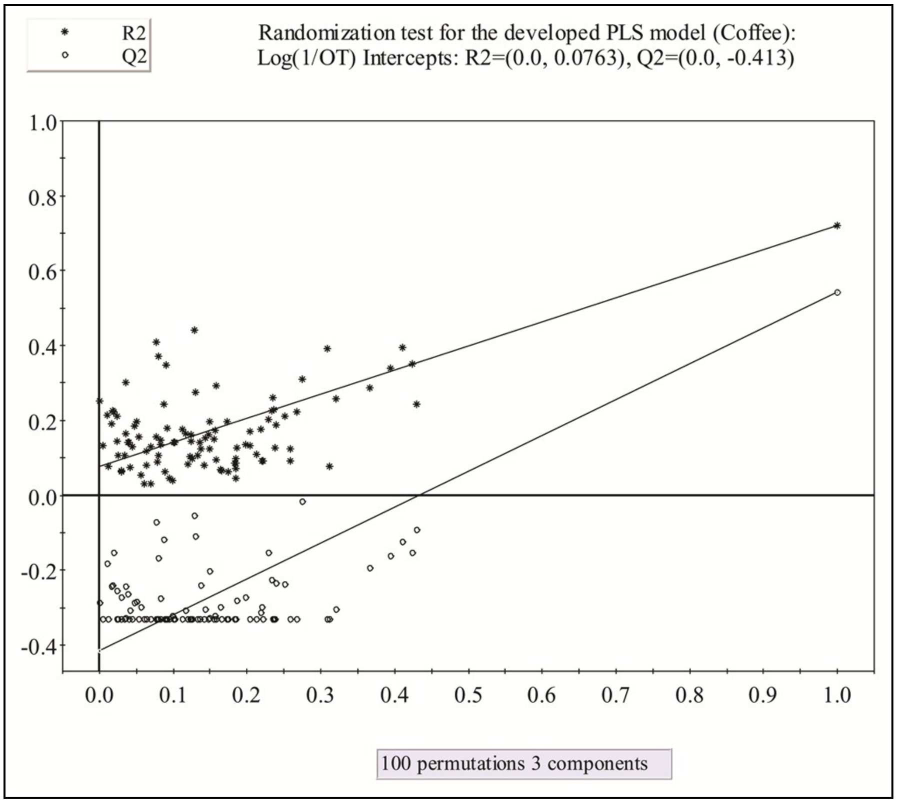

Fig. S2. The randomization plot for the QSPR model derived from PLS analysis using the odorants present in coffee.

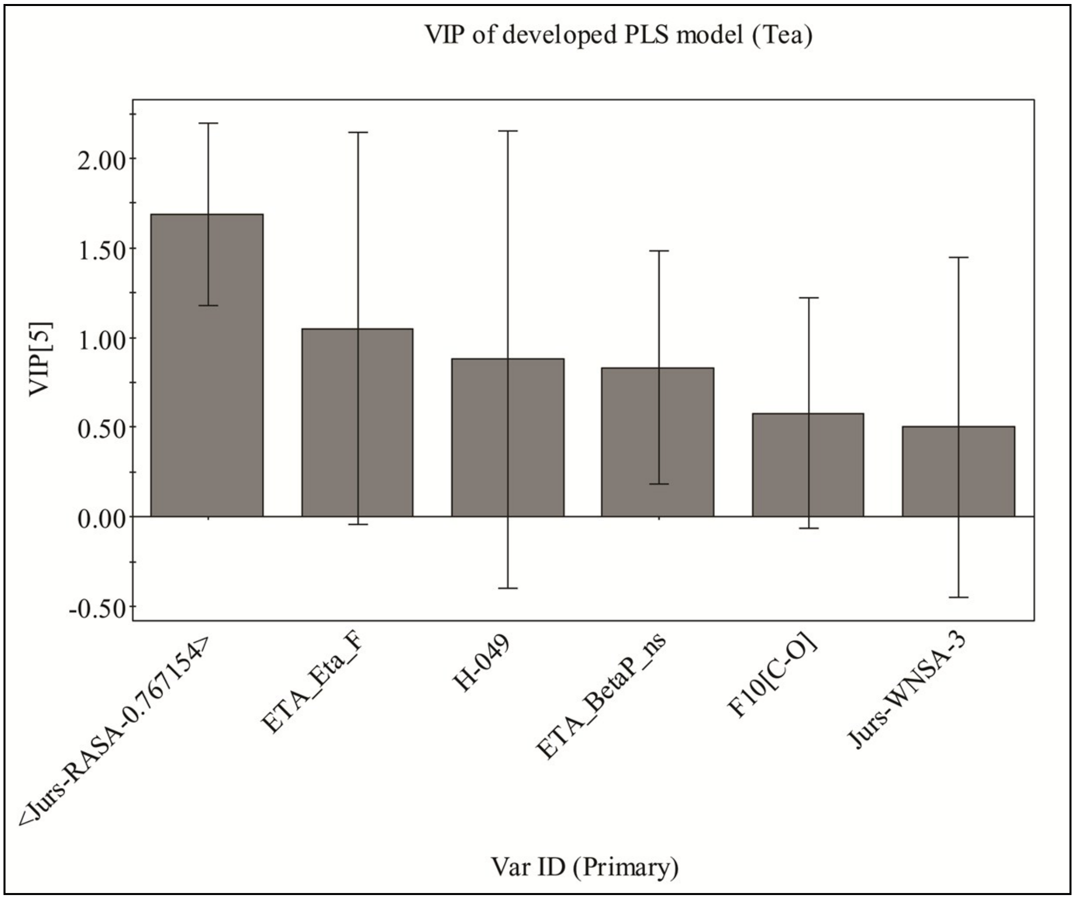

Fig.S3. Variable importance plot (VIP) for the final PLS model derived from the odorants present in black tea.

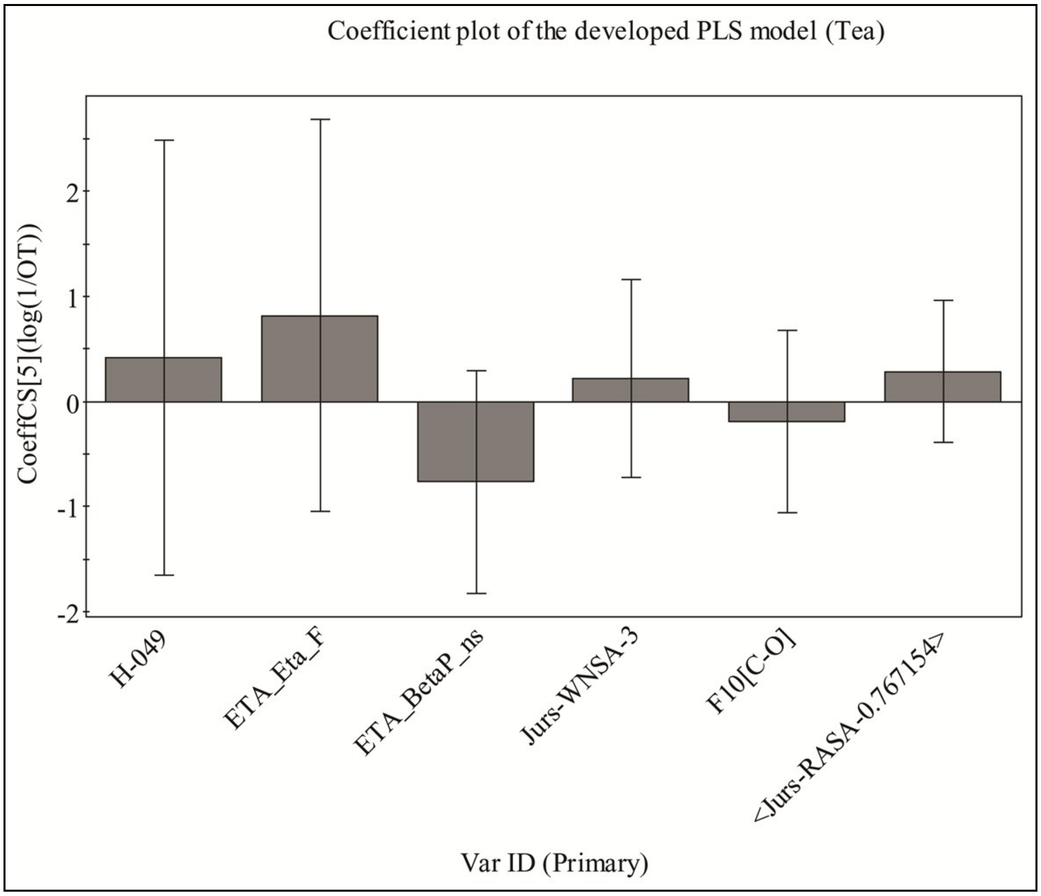

Fig. S4. Regression coefficient plot of the final PLS model derived from the odorants present in black tea.

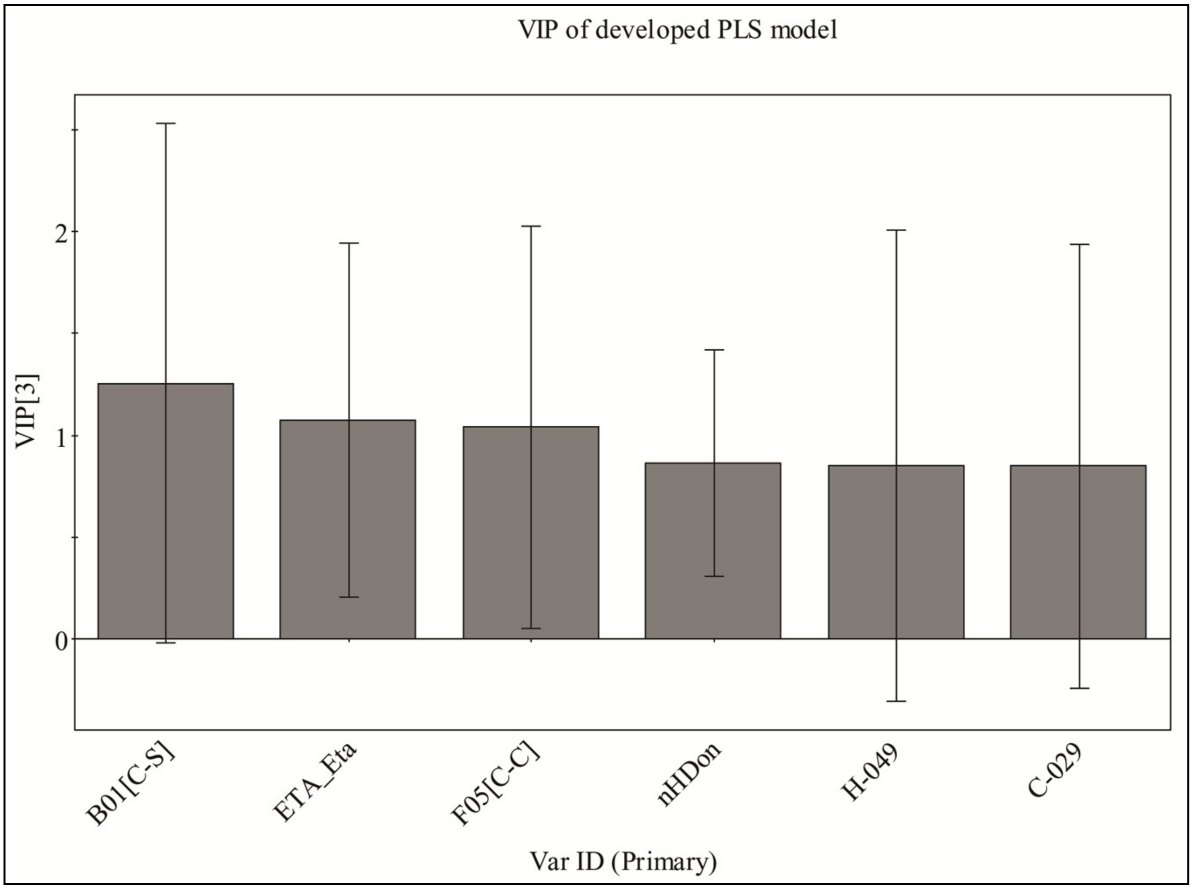

Fig.S5. Variable importance plot (VIP) for the final PLS model derived from the odorants present in coffee.

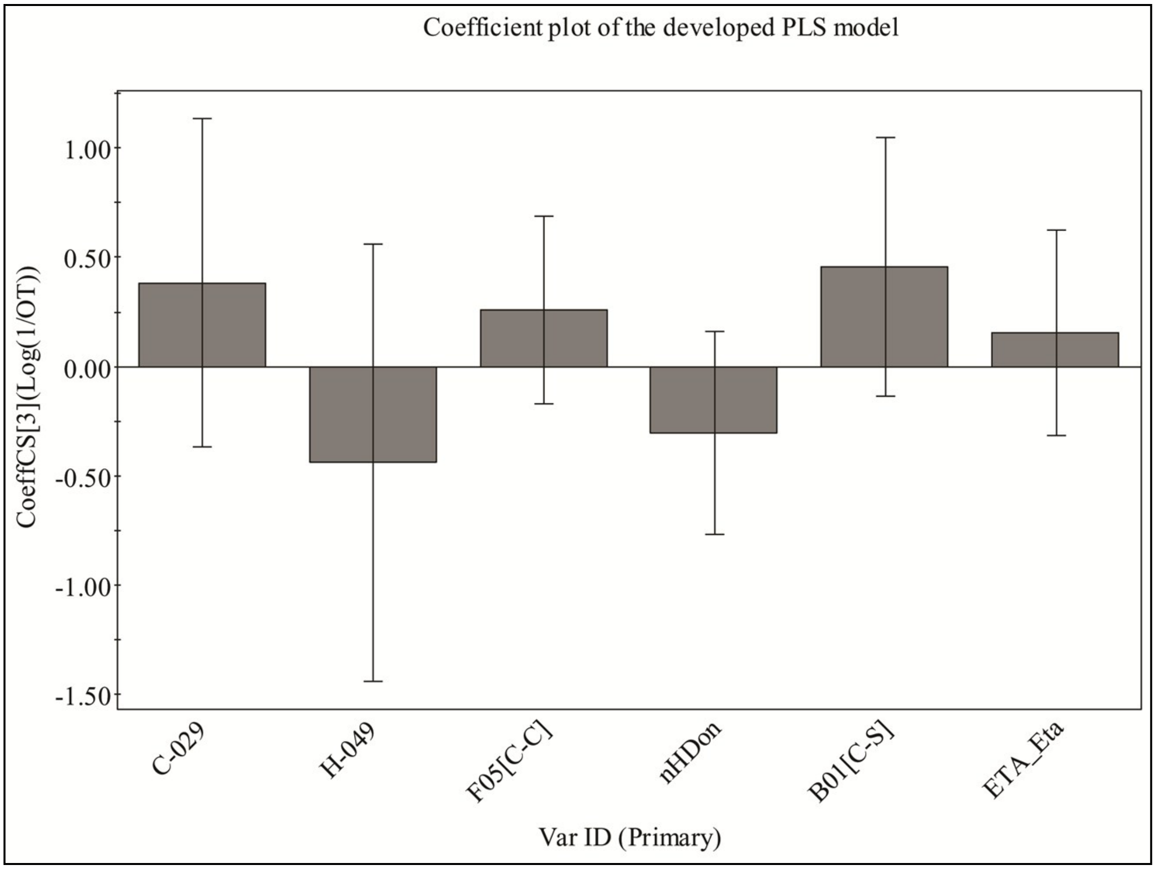

Fig. S6. Regression coefficient plot of the final PLS model derived from the odorants present in coffee.

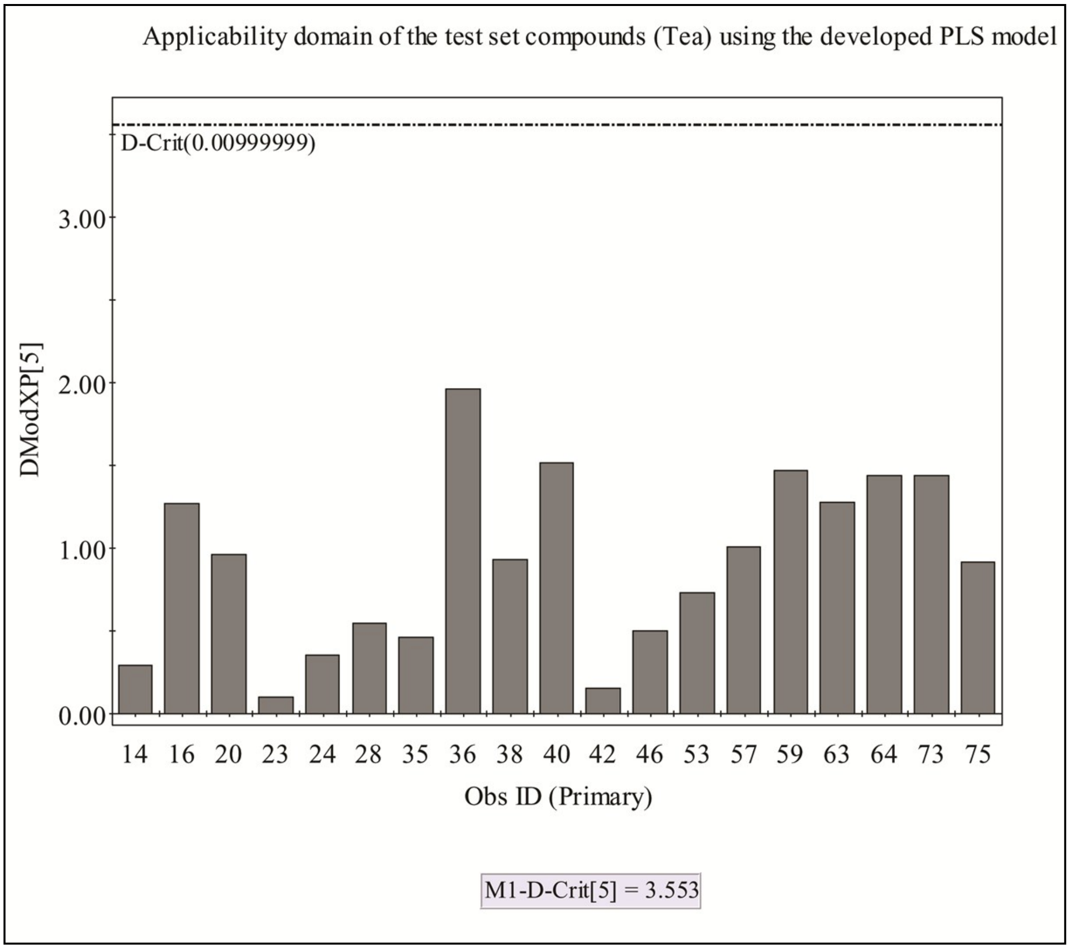

Fig. S7. DModX values of the test set compounds at 99% significance level for the developed PLS model derived from the odorants present in black tea. The dash horizontal line signifies the critical DModX value (3.553) at the 99%confidence level.

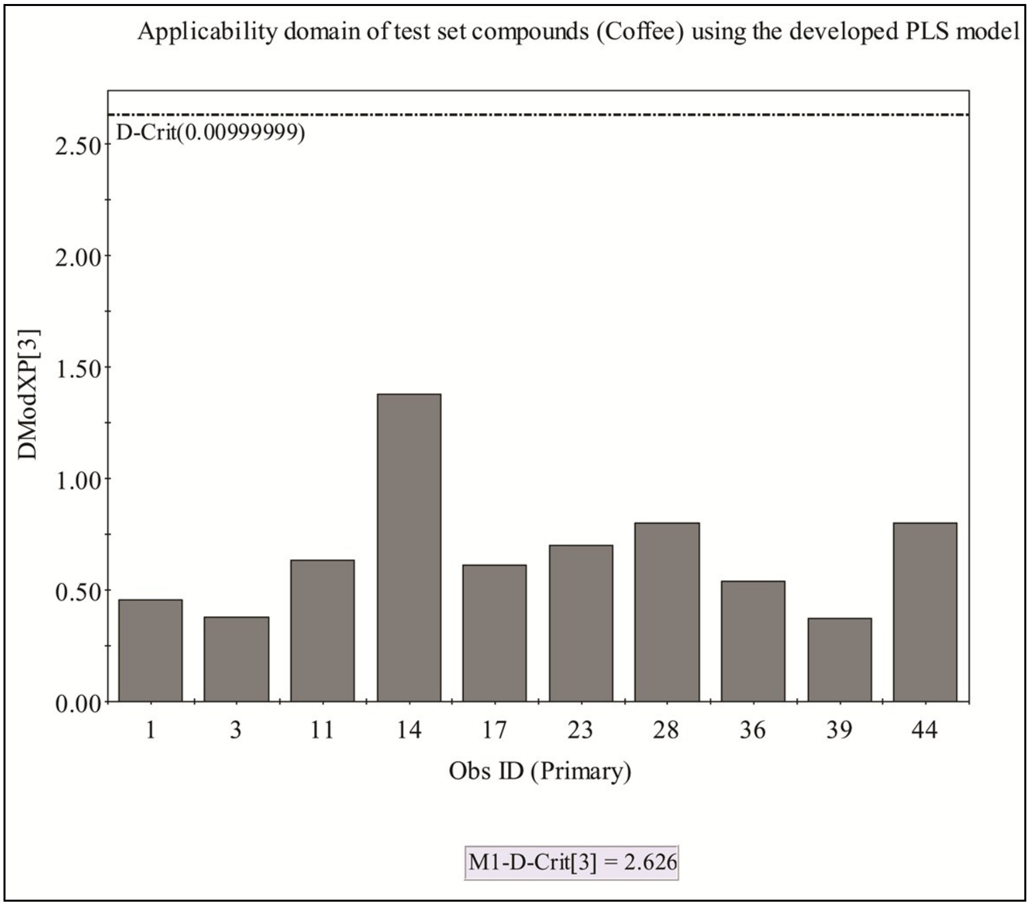

Fig.S8. DModX values of the test set compounds at 99% significance level for the developed PLS model derived from the odorants present in coffee. The dash horizontal line signifies the critical DModX value (2.626) at the 99%confidence level.
